# sncRNA-1 Is a Small Noncoding RNA Produced by *Mycobacterium tuberculosis* in Infected Cells That Positively Regulates Genes Coupled to Oleic Acid Biosynthesis

**DOI:** 10.3389/fmicb.2020.01631

**Published:** 2020-07-28

**Authors:** Fatma S. Coskun, Shashikant Srivastava, Prithvi Raj, Igor Dozmorov, Serkan Belkaya, Smriti Mehra, Nadia A. Golden, Allison N. Bucsan, Moti L. Chapagain, Edward K. Wakeland, Deepak Kaushal, Tawanda Gumbo, Nicolai S. C. van Oers

**Affiliations:** ^1^Department of Immunology, University of Texas Southwestern Medical Center, Dallas, TX, United States; ^2^Center for Infectious Diseases Research and Experimental Therapeutics, Baylor Research Institute, Baylor University Medical Center, Dallas, TX, United States; ^3^Tulane National Primate Research Center, School of Medicine, Tulane University, Covington, LA, United States; ^4^Texas Biomedical Research Institute, San Antonio, TX, United States; ^5^Department of Microbiology, University of Texas Southwestern Medical Center, Dallas, TX, United States; ^6^Department of Pediatrics, University of Texas Southwestern Medical Center, Dallas, TX, United States

**Keywords:** *Mycobacterium tuberculosis*, small RNAs, miRNAs, oleic acid, gene regulation

## Abstract

Nearly one third of the world’s population is infected with *Mycobacterium tuberculosis* (*Mtb*). While much work has focused on the role of different *Mtb* encoded proteins in pathogenesis, recent studies have revealed that *Mtb* also transcribes many noncoding RNAs whose functions remain poorly characterized. We performed RNA sequencing and identified a subset of *Mtb* H37Rv-encoded small RNAs (<30 nts in length) that were produced in infected macrophages. Designated as smaller noncoding RNAs (sncRNAs), three of these predominated the read counts. Each of the three, sncRNA-1, sncRNA-6, and sncRNA-8 had surrounding sequences with predicted stable secondary RNA stem loops. Site-directed mutagenesis of the precursor sequences suggest the existence of a hairpin loop dependent RNA processing mechanism. A functional assessment of sncRNA-1 suggested that it positively regulated two mycobacterial transcripts involved in oleic acid biosynthesis. Complementary loss- and gain- of-function approaches revealed that sncRNA-1 positively supports *Mtb* growth and survival in nutrient-depleted cultures as well as in infected macrophages. Overall, the findings reveal that *Mtb* produces sncRNAs in infected cells, with sncRNA-1 modulating mycobacterial gene expression including genes coupled to oleic acid biogenesis.

## Introduction

Tuberculosis (TB) is among the top 10 killers of humans (Murray and Lopez, 1996; Dheda et al., 2016; Furin et al., 2019; WHO, 2019). Estimates suggest that ∼2.3 billion individuals are currently infected with *Mycobacterium tuberculosis* (*Mtb*), with about 2,000 humans dying daily (Dheda et al., 2016; WHO, 2019). However, the majority of those people infected develop a life-long latency without progression to active TB, although there is evidence that some of the latency may actually reflect recently transmitted infections (Dheda et al., 2016; Houben and Dodd, 2016; Behr et al., 2018). The molecular processes regulating TB infection and pathogenesis remain incompletely understood (Cole et al., 1998; Supply et al., 2013). This is partly due to the complex nature of the 4.4 Mb *Mtb* genome, which has a high GC content >65%, roughly 4,000 open reading frames encoding proteins for which 1/3 have limited functional information (Cole et al., 1998; Cortes et al., 2013; Shell et al., 2015). Several of these genes have been characterized based on their ability to block phagolysosome fusion and processing in infected macrophages (Hmama et al., 2015). About 20% of the *Mtb* transcriptome comprises non-coding RNAs, including an abundance of small RNAs (sRNAs) (Arnvig and Young, 2009, 2012; DiChiara et al., 2010; Arnvig et al., 2011; Pellin et al., 2012; Cortes et al., 2013). These sRNAs, ranging in size from 50 to 350 nucleotides (nts), act as either translational activators or repressors (Arnvig and Young, 2009, 2012; Arnvig et al., 2011; Cortes et al., 2013; Solans et al., 2014; Gerrick et al., 2018). One stress responsive sRNA, called MrsI (ncRV11846), reduces the expression of non-essential iron-containing proteins by using a seed sequence to bind the 5′ untranslated region (UTR) of the mRNA target (Gerrick et al., 2018). A distinct sRNA, called Mcr11 (ncRv11264c), is required for *Mtb* growth in fatty acid depleted media, as it regulates targets involved in lipid production (Girardin and McDonough, 2020). This regulation was proposed mediated by a base-pairing between the sRNA and multiple transcripts. In a third study, 6C was identified as a sRNA that regulates genes involved in DNA replication and protein secretion via interactions using C-rich loops to complex mRNAs (Mai et al., 2019). Finally, PhoP positively regulates the expression of a 350-nt sRNA termed Mcr7 (Solans et al., 2014). A segment of Mcr7 binds to the mRNA for Twin arginine translocation (TatC), effectively occluding the ribosomal binding site (RBS).

The *Mtb* sRNAs so far described are >50 nts in length, with smaller RNAs of <50 nts yet to be reported. Such smaller RNA species do exist in other bacterial strains, where they function as both positive and/or negative regulators of gene expression (Grosshans and Filipowicz, 2008; Waters and Storz, 2009; Storz et al., 2011). These smaller RNAs have lengths as short as eukaryotic microRNAs (miRNAs), which are between 20 and 22 nts. However, unlike bacterial sRNAs which can act either positively or negatively, miRNAs primarily regulate mRNAs in a negative manner, determined by the seed-sequence base-pairing between the miRNA and most often, the 3′ UTR of a specific transcript (Bartel, 2009; Guo et al., 2010). To date, there are only two reports of miRNAs produced by mycobacteria (Furuse et al., 2014; Chakrabarty et al., 2019). *M. marinum* generates a single miRNA, with the precursor form of this miRNA containing the characteristic stem loop feature of eukaryotic precursor miRNAs (Furuse et al., 2014). While the *M. marinum* miRNA assembles with the eukaryotic RNA induced silencing complex (RISC), its targets remain unidentified. A second study reported on the existence of six distinct *Mtb*-encoded miRNAs, identified in serum samples from TB-infected individuals (Chakrabarty et al., 2019). The functional roles of these six *Mtb*-miRNAs are unknown, and they had an unusual feature in that many were comprised almost entirely of G and C nucleotides (Chakrabarty et al., 2019).

In the present study, we used RNA sequencing strategies to identify those sRNAs <30 nts generated in *Mtb*-infected macrophages. In addition to hundreds of mammalian miRNAs, 35 distinct *Mtb*-encoded smaller noncoding RNAs (sncRNAs) were identified. These increased in expression over the intracellular infection period. The majority of the sequence reads were in seven such sncRNAs. Three of them, sncRNA-1, sncRNA-6, and sncRNA-8 were thought to be derived from longer transcripts with putative precursor RNA sequences exhibiting predicted stable secondary structures with stem loops. Gain-of-function studies indicated that sncRNA-1 positively regulates genes required for oleic acid synthesis. When constitutively expressed, sncRNA-1 enhanced *Mtb* growth in nutrient-depleted conditions and enabled mycobacterial survival within infected macrophages. Targeting of sncRNA-1 with locked nucleic acid power inhibitors reduced mycobacterial survival in infected cells. Taken together, our findings suggest an important contribution of an *Mtb*-encoded sncRNAs in mycobacterial pathogenesis.

## Materials and Methods

### Ethics Statement

Mouse animal work described in this manuscript has been approved and conducted under the oversight of the UT Southwestern Institutional Animal Care and Use Committee (APN number 2015-101247). Mice were housed in a specific pathogen-free facility at UT Southwestern Medical Center.

### Bacteria

*Mycobacterium tuberculosis* H37Rv (ATCC #27294) was grown in 7H9 media supplemented with 10% oleic albumin dextrose catalase (OADC) (Remel, Thermo-Fisher) and 5% glycerol (G9012, Sigma- Aldrich). The auxotroph mutants of *Mtb* H37Rv 6206 (ΔpanCD, ΔleuC) and 6230 (ΔRD1, ΔpanCD) were kindly provided by Dr. William Jacobs (Sampson et al., 2004; Sambandamurthy et al., 2006). 6230 was grown in the 7H9 supplemented with 0.05% tyloxapol (T0307, Sigma-Aldrich), 0.2% casamino acids (223050, BD Bacto^TM^) and 24 μg/ml pantothenate (D-pantothenic acid hemicalcium salt, Sigma-Aldrich). The media for 6206 was similar to that used for 6230 with an additional supplement of 80 mg/ml leucine (Sigma-Aldrich).

### sncRNA Screening

For the screening of sncRNAs, the human-derived THP-1 monocyte/macrophage cell line, obtained from American Type Culture Collection (ATCC TIB-202^TM^), was used. The THP-1 cells were cultured in RPMI 1640 medium supplemented with 20% FBS (Hyclone or Atlanta Biologicals). Prior to infections, the cells were stimulated with PMA (final concentration 10^–9^ M) for 24 h to establish adherence to the plates. PMA activated THP-1 cells, either uninfected or 3- and 6-days post-infection with *Mtb* H37Rv (MOI = 10:1) were lysed in a Qiazol lysis buffer, followed by bead beating with glass beads. Total RNA was extracted with the Qiagen miRNeasy kit following the manufacturers’ instructions (Qiagen, Valencia, CA, United States). Samples were first treated with DNAse (Ambion-Thermo-Fisher). Where required, the RNA was concentrated with an RNA Pure and Concentrate kit (Zymo Research, CA, United States). RNA quality and quantity were determined using a NanoDrop 2000/2000c spectrophotometer and/or Bioanalyzer (Thermo Fisher Scientific Inc.). Five μg of total RNA was processed using a sRNA discovery platform using an Illumina high throughput sequencer (LC Sciences, Houston, TX, United States). A total of nine samples were sequenced; three uninfected cultures (ctl_D3), three from macrophages infected for 3 days (RV-THP_D3), and three samples from a 6-day culture (RV-THP_D6).

### Small RNA Library Construction

A sRNA library was generated from samples using the Illumina Truseq^TM^ Small RNA Preparation kit according to Illumina’s TruSeq^TM^ Small RNA Sample Preparation Guide (Catalog # RS-930-1012, Part # 15004197 Rev. B, January 2011). The purified cDNA library was used for cluster generation on Illumina’s Cluster Station and then sequenced on Illumina GAIIx following vendor’s instruction for running the instrument. Raw sequencing reads (40 nts) were obtained using Illumina’s Sequencing Control Studio software version 2.8 (SCS v2.8) following real-time sequencing image analysis and base-calling by Illumina’s Real-Time Analysis version 1.8.70 (RTA v1.8.70). The extracted sequencing reads were used in the standard data analysis. A proprietary pipeline script, ACGT101-miR v4.2 (LC Sciences), was used for sequencing data analysis (Li et al., 2010; Meyer et al., 2011; Wei et al., 2011). The raw reads were mapped to the human genome first (Supplementary Figure S1). Next, the sequences were mapped to miRNAs and pre-miRNAs with miRbase. Species screened included hsa, ptr, ppy, ppa, ggo, ssy, mml, mne, pbi, age, lla, sla, lca, mmu, rno, cgr, bta, oar, eca, oan, cfa, ssc, mdo, meu, and sha. The reads that did not map to the human genome (no hit in the first step) were subsequently mapped to the *Mtb* H37Rv, as well as to all the miRNAs species in that miRbase database. Criteria for identifying and annotating miRNAs was undertaken by LC Sciences, using more rigorous algorithms developed for plant miRNAs; i.e., (i) the miRNA/miRNA^∗^ sequences should be on opposite stem-arms that can form a duplex with 2-nt 3′ overhangs; (ii) ≤4 mismatched miRNA bases between the miRNA and the other arm of the hairpin, which includes the miRNA^∗^, and (iii) ≤1 asymmetric bulge and only ≤2 bases, especially in the miRNA/miRNA^∗^ region. The raw data has been uploaded to Gene Expression Omnibus (GEO) with the accession number GSE146228.

### Copy Number Normalization for Small RNA Sequencing Data Analysis

A modified global normalization is used to correct copy numbers among different samples assuming that there is a subset of sequences that do not change significantly across all samples and in each sample the reading variations occur in the same proportion. First, a common set of sequences was found among all samples. Then, a reference data set was constructed. Each data in the reference set was the copy number median value of a corresponding common sequence of all samples. A 2-based logarithm transformation on copy numbers of all samples and reference data set was done and the differences between individual sample and the reference data set was calculated. A subset of sequences selected with a cut off of less than fourfold change from the reference set was formed. Linear regression between individual samples and reference set on the subset sequence was performed to derive linear equations such that *y = a x* + *b* where *a* and *b* are the slop and interception, respectively, of the derived line, *x* is log2 (copy #) of the reference set, and *y* is the expected log2(copy #) of the sample on a corresponding sequence. Mid values of the reference set were calculated. The corresponding expected log2(copy #) of a sample was calculated. Logarithmic correction factor of a sample was determined. Then, the arithmetic correction factor was derived. Copy numbers of individual samples were corrected by multiplying corresponding arithmetic correction factor to original copy numbers.

### Comparisons for Multiple RNA Sequencing Reads

For comparisons of multiple samples derived from the sRNA sequencing screen, raw reads of multiple samples were combined. The number of read copies from each sample was tracked during mapping and normalized for comparison. Reads may be mapped to multiple entries of the reference database and the number of the read copies was divided by the number of mapped entries. Normalization of sequence counts in each sample (or data set) was achieved by dividing the counts by a library size parameter of the corresponding sample. The library size parameter was a median value of the ratio between the counts a specific sample and a pseudo-reference sample. A count number in the pseudo-reference sample was the count geometric mean across all samples.

### sncRNA Structure Prediction and Sequence Alignment

The RNAFold web server was used for minimum free energy (MFE) secondary RNA structure predictions. The Institute for Theoretical Chemistry at the University of Vienna maintains this website^1^. sncRNA-1 sequence alignments between different mycobacteria species were done using SnapGene 5.0.6 software (GSL Biotech).

### Infection Assays

PMA activated THP-1 cells or bone marrow derived macrophages (BMDMs) were used for the infection assays. THP-1 cells were prepared as described in the RNA screening described above. BMDM were obtained from the femur, tibia, and spinal column of the C57BL/6 mice. Macrophages were differentiated using macrophage media, 30% of L929 supernatant (ATCC CCL-1^TM^) mixed with 70% of RPMI containing 10% FCS (Weischenfeldt and Porse, 2008). Briefly, red blood cells were lysed and the remaining cells cultured in macrophage media overnight. Non-adherent cells were collected and cultured for 4 days in macrophage media. On day 4, cells were detached from the culture plates and stained with an antibody detecting CD11b (Catalog # ab24874, Abcam). After confirming positive staining for the CD11b marker, the BMDMs were plated for infection experiments in RPMI media containing 10% FCS. Multiplicity of infections (MOI) with wild-type *Mtb* H37Rv and the various auxotroph mutants ranged between 1:1 and 10:1, as described in the figure legends. Three hours post-infection, the THP-1 macrophages or BMDMs were washed and cultured for indicated time courses. The RNA samples used for quantitative RT-PCR were isolated as described in the section “sncRNA Screening.” Custom designed locked nucleic acid miRNA primers were used to detect the sncRNAs with a quantitative miRNA RT-PCR assay and the miRCURY^TM^ system (Exiqon Inc., now part of Qiagen Inc.). For colony forming units (CFU) enumeration, the infected cells were lysed in PBS containing SDS (0.05% v/v). Ten-fold serial dilutions were made in the same buffer, and samples were spread on 7H10 agar plates containing 10% OADC and other supplements required for the auxotroph mutants to determine the total bacterial burden.

### Locked Nucleic Acid Power Inhibitor Assays

A locked nucleic acid power inhibitor (LNA-PI) targeting *Mtb*-encoded sncRNA-1 was designed and synthesized by Exiqon Inc., based on their proprietary nucleotide modifications (Exiqon Inc., Woburn, MA, United States, now part of Qiagen Corp.). A control LNA that does not target any known eukaryotic gene was included as a control (acgtctatacgccca). A fluorescent tag was incorporated at the 5′ end of the inhibitor to monitor uptake. LNA-PI against sncRNA-1 (350 nM final concentration) was used to stain *Mtb* H37Rv 6230 or 6206 by incubating the cells for 24 h. Stained bacteria were fixed in 2% PFA and fluorescence was detected with flow cytometry. For CFU experiments, BMDMs were infected at a MOI of 1:1 or 3:1. Infected BMDMs were lysed in 0.05% SDS at different time points post infection and plated on 7H10 plates.

### Cloning

*Mtb* H37Rv genomic DNA was obtained from Colorado State University under a TB Vaccine Testing and Research Materials Contract with the National Institutes of Health (N01-AI-40091). pKA-303 was used as an expression vector for the sncRNAs. It originally had a kanamycin resistance cassette which was replaced with a hygromycin resistance cassette because pKA-303 with kanamycin resistance cassette could not be propagated in *Mtb* H37Rv 6230. Primers are listed in Supplementary Table S5. Subcloning was done using NEBuilder^®^ HiFi DNA Assembly Master Mix (E2621S, NEB Inc.) according to the manufacturer’s instructions. Primer design was done using NEBuilder Assembly^2^ tool v2.2.5. DNA sequences encompassing the genomic locations of the individual sncRNAs were cloned into the modified pKA-303. Plasmid transformations were first done with chemically competent *Escherichia coli* (DH5alpha). DNA sequencing confirmed sequence insertion and orientation. Plasmids were then electroporated into *Mtb* H37Rv 6230 as described elsewhere (Eitson et al., 2012). Transformants were selected in the presence of hygromycin, used at a final concentration of 100 μg/ml. RNA was extracted from individual clones and sequenced at The Genomics and Microarray core at UT Southwestern Medical Center. The data is available in the GEO repository (GSE146228). KEGG GO term analysis was done using DAVID 6.8 available online^3^. Remaining RNA was used to quantify mature and precursor sncRNA expression. sncRNA expression was quantified using the miRNA RT-PCR assay as described before. Precursor sncRNA expression was quantified using high-capacity cDNA reverse transcription kit (ThermoFisher Scientific Inc.) and SYBR green PCR master Mix (ThermoFisher Scientific Inc.). Rv0242c was cloned into a protein expression vector called pSUM-Kan-MCS1-gfp (Eitson et al., 2012). The kanamycin cassette was replaced with a zeocin resistance cassette while GFP was replaced with the Rv0242c gene along with its 5′ UTR. Subcloning was done using NEBuilder^®^ HiFi DNA Assembly Master Mix. Plasmids expressing control vector or sncRNA-1 and Rv0242c were electroporated into *Mtb* H37Rv 6230 in combination. Transformants were selected in the presence of 100 μg/ml hygromycin and 100 μg/ml zeocin. RNA was isolated and Rv0242c expression was quantified with a quantitative RT-PCR assay, using the 16S rRNA as an RNA control.

### Site Directed Mutagenesis

For site directed mutagenesis, primers were designed using NEBasechanger^TM^ tool. The proposed binding site in the 5′ UTR of Rv0242c in the expression vector (pSUM-Zeo-Rv0242) was PCR amplified with primers designed for site directed mutagenesis. PCR product was treated with KDL mixture (NEB cat #0554) for 30 min 37°C and transformed into *E. coli* DH5alpha. Eight clones were randomly selected and propagated. Plasmids were isolated with ZymoPURE^TM^ Plasmid Miniprep Kit (Cat #D4209, Zymo Corp.) and sequenced by the McDermott Sanger Sequencing Core at UT Southwestern Medical Center. The plasmids with the desired mutations were electroporated to *Mtb* H37Rv 6230. Three clones were selected and propagated. RNA was extracted and Rv0242c expression was quantified using quantitative RT-PCR assay relative to 16S rRNA.

### *Mtb* Growth and Gene Expression

*Mtb* H37Rv 6230 expressing the control vector or sncRNA-1 vector was grown in 7H9 supplemented with 5% glycerol or with 5% glycerol and 10% OADC. Optical density at 600 nm was measured in 96 well plates in triplicates every 24 h for 5 days. After 5 days, RNA was extracted as described above and sncRNA-1 expression was quantified using the miRNA RT-PCR assay. Gene expression differences were determined with an associative *t*-test, as described (Dozmorov and Centola, 2003; Dozmorov and Lefkovits, 2009; Dozmorov et al., 2011). Ingenuity Pathway analyses were performed as previously described (Du et al., 2019).

### Northern Blotting

*Mtb* H37Rv 6230 clones expressing the control vector or sncRNA-1 vector were grown in 7H9 media containing the supplements for 1 week. RNA was extracted as described in the section “sncRNA Screening” above. Five μg RNA was resolved on a 20% acrylamide gel (29:1) for 2–3 h in a 1× Tris-cl, borate, EDTA buffer (TBE). The RNA bands were then transferred to Nylon N^+^ membranes at 80 volts for 1 h in 0.5× TBE buffer. RNA was UV crosslinked and the membrane was prehybridized at 39°C for 1–2 h in rapid-hybridization buffer (GE/Amersham). These conditions were fully described elsewhere (Belkaya et al., 2011). It was hybridized overnight at 39°C with Starfire probe specific for sncRNA-1 or 5S rRNA (Starfire^TM^, Idt DNA technologies, Inc.). The 5S probe detects a band near 120 bp. The membrane was washed twice in buffer (2× SSC buffer with 0.1% SDS), wrapped in saran wrap, and imaged with a phosphoimager.

### Statistical Analyses

Statistical tests used for each analysis is stated in respective figure legends. Statistical significance was indicated by the values and/or one (^∗^*p* < 0.05), two (^∗∗^*p* < 0.005), or three asterisks (^∗∗∗^*p* < 0.0005). Precise statistical values were provided in the figures, as shown.

## Results

### *Mtb* H37Rv Produces Many Smaller Noncoding RNAs During Intracellular Infections in Macrophages

**FIGURE 1:**
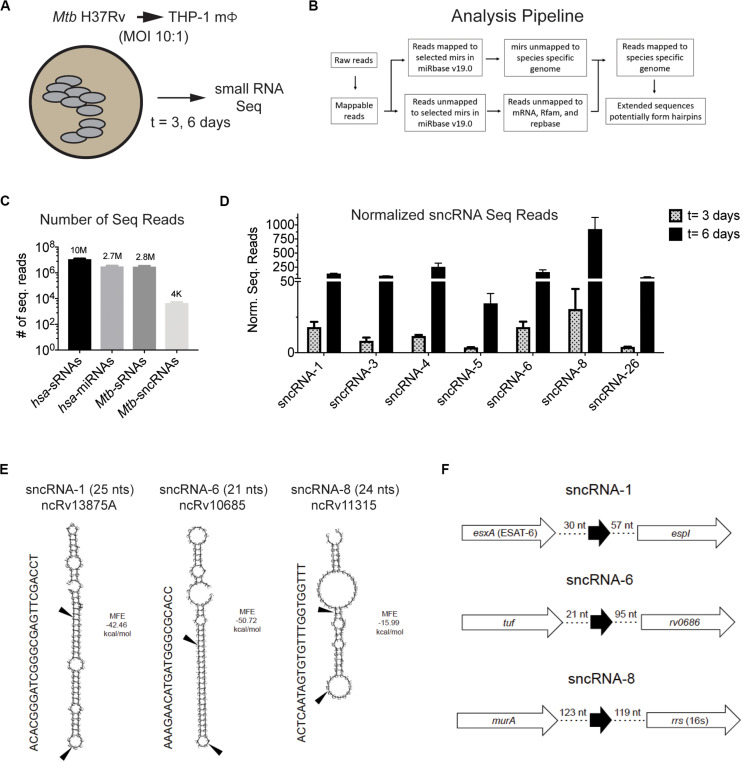
*Mtb* H37Rv produces many smaller noncoding RNAs during intracellular infections in macrophages. **(A,B)** Strategy to identify sncRNAs in *Mtb* is shown. **(A)** THP-1 macrophages were infected with *Mtb* H37Rv (10:1) for 3 h and RNA was isolated from THP-1 infected for 3 and 6 days. **(B)** RNA Seq was applied to sRNAs using the given algorithm. **(C)** The normalized read counts of sRNAs and miRNAs that mapped to the eukaryotic and mycobacterial genome are indicated. The *Mtb*-encoded sRNAs that resembled miRNAs were designated as sncRNAs. **(D)** The normalized sequence reads for the seven most abundant sncRNAs were determined at 3- and 6-days post-infection. Mean ± SD were determined from triplicate samples/time point, with *p*-values ranging from 6 × 10^–3^ to 1 × 10^–10^. **(E)** Secondary structure of sncRNA-1, sncRNA-6, and sncRNA-8 precursors predicted with RNAfold are shown. MFE < 0 indicates stable RNA structure. The mature forms of the sncRNAs are indicated with arrows. Other designation of these sncRNAs are shown below the titles. **(F)** Genomic positions of sncRNA-1, sncRNA-6, and sncRNA-8 are shown.

RNA was isolated from uninfected THP-1 macrophages and from those infected with *Mtb* H37Rv (MOI = 10:1) at days 3 and 6 post-infection and sRNAs were sequenced (Figure 1A). Data analysis was performed to detect novel sRNAs of <30 nts (Figure 1B and Supplementary Figure S1). Briefly, the normalized sequencing reads were first mapped to human genome (HumanRefSeqBuilt37; GRCh37p13), with 13.5 and 7.1 million human-encoded sRNA sequence reads identified pre- and post- infection, respectively (Supplementary Figure S1). This filtering was further refined for miRNA-like sRNAs, as detailed in the section “Materials and Methods.” The selection criteria included sRNAs ≤30 nts that had surrounding sequences in the genome with significant potential for secondary RNA structures, including stem loops. Using this strategy, 15 and 30% of the sRNAs sequenced pre- and post-infection, respectively, were classified as human miRNAs (*hsa*-miRNAs) (Figure 1C). A total of 2,034 *hsa*-miRNAs were identified, 1,028 in the 5p direction and 1,006 in the 3p orientation. By selecting miRNAs with a read number greater than the data set average (2,995), 48 *hsa*-miRNAs were significantly up- and/or down- regulated during the infection (Supplementary Figure S2). Target prediction programs suggested that these miRNAs affected genes coupled to the immune system, metabolic processes, cell communication, and developmental events (data not shown). These findings are consistent with previous reports wherein many miRNAs are up- and down-regulated in mycobacterium-infected eukaryotic cells (Kumar et al., 2012; Wu et al., 2012; Dorhoi et al., 2013; Miotto et al., 2013; Singh et al., 2013; Furuse et al., 2014; Ni et al., 2014; Chakrabarty et al., 2019).

The sRNAs that did not map to a human sequence were screened against the *Mtb* H37Rv genome (NC_000962). Many *Mtb*-encoded sRNAs were identified, consistent with previous reports detecting sRNAs in diverse mycobacterial species (Supplementary Figure S1C) (Arnvig and Young, 2009, 2012; Arnvig et al., 2011; Pellin et al., 2012; Gerrick et al., 2018). Of the sRNAs detected, only 0.15% had a resemblance to miRNAs. These were smaller than 30 nts with secondary structures of putative precursor RNA sequences characteristic of mammalian and/or plant miRNAs, particularly stem loops. Thirty-five distinct *Mtb*-encoded sRNA (<30 nt) meeting these criteria were identified and herein referred to as sncRNAs (Figure 1C). They were designated sncRNA-1 to sncRNA-35 based on their descending *p*-values (Table 1 and Supplementary Table S1). The sncRNAs size ranged from 18 to 30 nts with an average GC content of 50% (Supplementary Table S1).

**TABLE 1:** Identification and genomic location of seven distinct *M. tuberculosis*-encoded smaller noncoding RNAs produced in infected human macrophages.

**sncRNA designation^a^**	**Nucleotide sequence**	**Length**	**Genomic location^b^**	**Nearby coding sequence^c^**	**Other designations**
sncRNA-1^d^	ACACGGGAUCGGGCGAGUUCGACCU	25	4352927–4352951	b/w^f^ esxA (Rv3875) and Rv3876	ncRv13875A
sncRNA-3	ACGGGCAGACUAGAGUACUGCAGGGGAGAC	30	1472478–1472507	rrs (Rvnr01)	ncRv0001
sncRNA-4	GAAAUGACGCAAUGACCUCU^e^	20	3481440–3481459	Rv1199c and b/w Rv1200	ncRv11199A
sncRNA-5^d^	CGUUUCGAAGGAUCACGCGAUGACCGCCC^e^	29	3481390–3481418	b/w Rv1199c and Rv1200	ncRv11199B
sncRNA-6^g^	AAAGAACAUGAUGGGCGCACC^e^	21	786003–786083	b/w iron elongation factor (Rv0685) and (Rv0686)	ncRv10685
sncRNA-8^d^	ACUCAAUAGUGUGUUUGGUGGUUU^e^	24	1471701–1471724	b/w murA (Rv1315) and rrs (Rvnr01)	ncRv11315
sncRNA-26	CGGCAACUGAAUACUGACC	19	4075666–4075684	b/w Rv3190c and Rv3191c	ncRv13190

*^a^sncRNA number determined by p-value significance, calculated from n = 3/time point. ^b^Location on the Mtb H37Rv genomic sequence. ^c^Sequence on or near coding sequences. ^d^sncRNAs (sncRNA-1, sncRNA-5, sncRNA-8, sncRNA-19, and sncRNA-26) detected in TB infected human and/or monkey lung tissue. ^e^Sequences independently detected in Mtb-infected macrophages with an initial pilot small RNA discovery assay that was performed. ^f^b/w = between the indicated coding proteins. ^g^sncRNA-6 and a smaller homologous 19-nt species are present in multiple locations in the genome.*

The changes in the expression of the seven most abundant sncRNAs are shown in Figure 1D. As this screen was initially designed to identify *hsa*-encoded small regulatory RNAs, the expression of *Mtb*-encoded sncRNAs independent of infection had not been assessed with the RNA-seq approach. Even so, the expression of sncRNAs increased from day 3 to 6 post infection, revealed by the normalized RNA-seq reads (Figure 1D). The seven sncRNAs identified were distributed throughout the *Mtb* genome, with most contained in intergenic regions, and several mapped at multiple locations, including sncRNA-4, sncRNA-5, sncRNA-6, and sncRNA-26 (Table 1 and Supplementary Table S1). sncRNA-1 and sncRNA-28, separated by 32 bases, along with sncRNA-22 and sncRNA-30, were near the genes encoding virulence factors, including *esxA*, *esxB*, and Rv3876 (Supplementary Table S1). sncRNA-3, sncRNA-8, sncRNA-16, and sncRNA-33 clustered between Rvnr01 and Rv1315, ending at the coding sequences of a ribosomal RNA and *murA* (Supplementary Table S1). Supporting our findings, previous transcriptomic screens uncovered several longer noncoding RNA transcripts in which sncRNA-6 (ncRv10685), sncRNA-8 (Mcr3), sncRNA-17 (MTS1082), sncRNA-19 (Mcr11), and sncRNA-21 (Mcr14) are contained (Arnvig and Young, 2009; DiChiara et al., 2010; Arnvig et al., 2011).

Of the 35 sncRNAs identified, sncRNA-1, sncRNA-6, and sncRNA-8 were analyzed in more detail as they predominated the sequence reads and had smaller negative MFE values reflecting stable RNA structures (Figures 1D,E). sncRNA-1 resides between *esxA* and *espI*, genes within the major pathogenicity locus for *Mtb* (RD1) (Figure 1F) (Brosch et al., 2002; Marmiesse et al., 2004). The 21-nt sncRNA-6 is located at two sites, including one between *tuf*, an iron-regulated elongation factor and *Rv0686*, involved in cell wall biogenesis (Figure 1F) (Zvi et al., 2008). There are additional sncRNA-6 homologous sequences that are 19 nt in length, and lack 2 nt in the 3′ end of the mature sncRNA-6 (data not shown). sncRNA-8 is located between *murA* and rrs (16s RNA), with *murA* involved in cell wall formation (Figure 1F) (Babajan et al., 2011). Whether the sncRNAs present at multiple loci were expressed from each location has not been evaluated.

### The Levels of Three Distinct *Mtb*-Encoded sncRNAs Increase During the Course of an Infection

**FIGURE 2:**
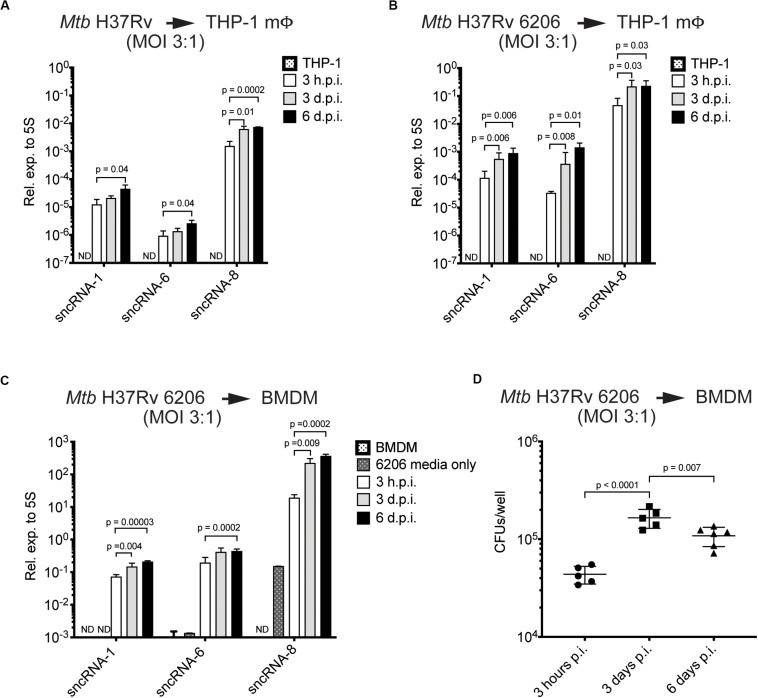
The levels of three distinct *Mtb*-encoded sncRNAs increase during the course of an infection. **(A–C)** Quantitative miRNA RT-PCR was performed on RNA extracted from THP-1 macrophages **(A,B)** or BMDMs **(C)** infected with *Mtb* H37Rv The *Mtb* strains included *Mtb* H37Rv **(A)** and an auxotroph mutant, *Mtb* H37Rv 6206 (ΔpanCD, ΔleuCD) **(B,C)**. miRCURY locked nucleic acid (LNA^TM^) modified primers selective for sncRNA-1, sncRNA-6, and sncRNA-8 were synthesized, along with a primer pair to detect mycobacterial 5S RNA, which was used for normalization purposes. The analysis was done with samples obtained 3 h, 3-days, and 6-days post-infection, using initial MOIs of 3:1. RNA was isolated from *Mtb* H37Rv 6206 incubated in the supplemented BMDM culture media for 3 h as a negative control in 6206 media only on panel **(C)**. *p*-values were calculated using one-way ANOVA. *p*-values designated as significant (<0.05) are shown. ND designates not detected. Log scale was used to show the expression of three distinct sncRNAs. **(D)** CFU/well burden in infected BMDMs was determined 3- and 6-days post infection using initial MOIs of 3:1. Cells were lysed in 0.05% SDS and serially diluted before plating on supplemented 7H10 agar plates. Experiments in panels **(C)** and **(D)** were done in parallel. Shown are a representative image of two independent experiments.

To assess how the levels of the sncRNAs change over time in infected cells, the miRCURY^TM^ locked nucleic acid (LNA)^TM^ quantitative miRNA PCR system was used. This is a highly sensitive and specific assay initially developed for quantitating miRNAs (Petersen et al., 2002; Stein et al., 2010). RNA was isolated from THP-1 macrophages or BMDMs, either uninfected or infected with either the virulent strain *Mtb* H37Rv, or the *Mtb H37Rv 6206 auxotroph* mutant strain (MOIs = 3:1) at multiple time points. The *Mtb* H37Rv 6206 auxotroph strain lacks panCD and leuC in its genome but has similar growth and infectivity characteristics as the parental *Mtb* H37Rv strain when the media is supplemented with pantothenate and leucine (pan^+^ leu^+^ media) (Sampson et al., 2004). Moreover, it only requires BSL2 plus biosafety conditions. sncRNA-1, sncRNA-6, and sncRNA-8 were consistently detected in the various mycobacteria used. All three sncRNAs increased in expression between 3- and 30-fold during the 6-day infection period (Figures 2A–C). These three sncRNAs were not detected in uninfected macrophages, although some sncRNA-6 signal was found to be just above background levels in BMDMs. To determine whether sncRNAs were produced independent of any infection, their levels were compared with the *Mtb* H37Rv 6206 auxotroph grown for 3 h in the leu^+^ pan^+^ macrophage media and harvested at the same time point as the cultures used for intracellular infections. sncRNA-1 and sncRNA-6 were not detected in the media only control, while a basal level of sncRNA-8 was noted (Figure 2C). sncRNA-8 increased 100-fold within 3 h post-infection in macrophages (Figure 2C).

The increasing levels of sncRNA-1, sncRNA-6, and sncRNA-8 detected in infected cells was revealed with two independent assays, quantitative miRNA PCR assays and RNA-seq reads (Figures 1D, 2A). However, the sncRNA levels appeared to be higher with RNA-Seq, which is partially explained by the higher MOIs used in the initial screen (10:1). Interestingly, the expression levels of all three sncRNAs was also higher in BMDMs versus THP-1 cells, suggesting a cell type dependent effect on their induction or processing (Figures 2A,C). Finally, the number of CFU of *Mtb* H37Rv 6206 dropped by day 6 relative to day 3 while the sncRNA levels still increased (Figures 2C,D). We also attempted to use a Northern blotting to detect the sncRNA. For these experiments, RNA was isolated from BMDMs in pan^+^ leu^+^ media following infections at a MOI of 10:1 using *Mtb* H37Rv 6206. There was no appreciable signal for sncRNA-1 in the Northern blots (data not shown). When probing the same membrane for the more highly expressed *Mtb* 5S RNA, only a very weak signal was found after overnight exposures (Supplementary Figure S3). These experiments indicate that Northern blotting procedures are not sensitive enough to detect the endogenous sncRNAs unless they are over-expressed (see later sections). The detection of these sncRNAs is also hampered by the over-riding abundance of the mammalian RNAs. Thus, for our probing studies, we relied on the miRCURY^TM^ locked nucleic acid (LNA)^TM^ quantitative miRNA PCR system.

### *Mtb* sncRNA-1 Positively Regulates the Fatty Acid Metabolism

**FIGURE 3:**
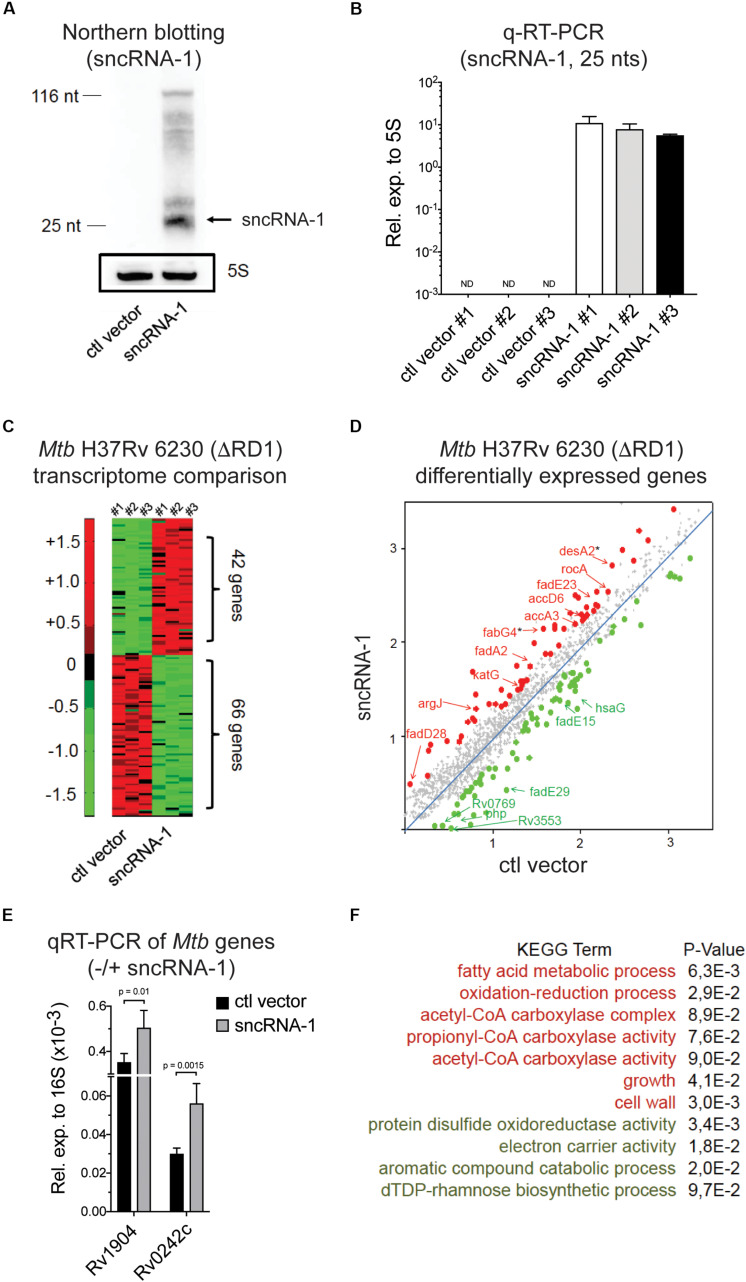
*Mtb* sncRNA-1 positively regulates the fatty acid metabolism. **(A)** sncRNA-1 was cloned into the pKA-303 RNA expression vector. The pKA-303-sncRNA-1-hygromycin containing plasmid or the empty vector were electroporated into *Mtb* H37Rv 6230 (ΔRD1, ΔpanCD) and individual clones were isolated and propagated. RNA was extracted from three clones/group at the stationary phase. sncRNA-1 expression was visualized with Northern blotting using a sncRNA-1 specific probe in clones expressing either the control vector or sncRNA-1 expression vector. A total of 25 nts mature form of sncRNA-1 is indicated with the arrow. The same blot was probed for 5S RNA which is shown in the box. Shown is a representative image of three clones. **(B)** sncRNA-1 was quantified using miRCURY LNA^TM^ quantitative miRNA PCR system relative to mycobacterial 5S rRNA. **(C)** RNA was extracted from the indicated clones and used for RNA Seq. A representative heat map, using a 1.5-fold differential expression cutoff, illustrates the differential gene expression (red = elevated expression; green = low expression). **(D)** Differentially regulated bacterial transcripts are shown. Ones highlighted with a star are those identified as putative sncRNA-1 targets. **(E)** These putative targets were quantified in *Mtb* H37Rv 6230 clones expressing either the ctl vector or the sncRNA-1 expression vector with qRT-PCR relative to 16S rRNA. **(F)** Using significantly up/down regulated genes in DAVID 6.8, KEGG GO term analysis was performed. Pathways for which *p* < 0.1 are shown.

Of the three sncRNAs that had the greatest similarity to miRNAs, we initially selected sncRNA-1 for follow-up functional studies. There were several reasons for selecting sncRNA-1 in our studies. First, nothing has been reported in the literature about sncRNA-1. While neither sncRNA-6 or sncRNA-8 were previously identified, longer RNA transcripts containing these two noncoding RNAs were previously described (DiChiara et al., 2010). Second, sncRNA-1 resides within RD1, which is the principal pathogenicity region in *Mtb*. Thus, sncRNA-1 could have a role in mycobacterial pathogenesis. Neither sncRNA-6 or sncRNA-8 were present in defined pathogenicity loci. Third, auxotroph mutants of *Mtb* H37Rv were already available [*Mtb* H37Rv 6230 (ΔRD1, ΔpanCD)] that lacked sncRNA-1 due to the loss of the RD1 locus. This enabled us to use a gain-of-function approach with this auxotroph mutant in the absence of the endogenous transcript. A second advantage is that *Mtb* H37Rv 6230 has a faster doubling time (3–4 h) than *Mtb* H37Rv and can be used in BSL2 Plus conditions (Sampson et al., 2004; Sambandamurthy et al., 2006; Volpe et al., 2006). For the gain-of-function experiments, a longer DNA segment (208 nts) that contained sncRNA-1, which included the hairpin loop structure, was cloned into an RNA expression vector pKA-303 (Arnvig et al., 2011). This vector uses the *rrnB* promoter to drive RNA expression and was used since the endogenous regulatory elements that control sncRNA-1 are not known (Supplementary Figure S4). The plasmids were electroporated into *Mtb* H37Rv 6230, and multiple antibiotic resistant clones were expanded and screened. Northern blotting revealed that the clones overexpressing the DNA segment containing sncRNA-1 generated a processed form of sncRNA-1, with a size corresponding to 25 nts (Figure 3A). Most of sncRNA-1 was processed, but some larger sized RNA transcripts that ranged between 25 and 116 nts were detected (Figure 3A). The same blot was probed for 5S RNA which revealed comparable RNA loading (Figure 3A). Using three independent sncRNA-1 transformants, RNA was extracted, and the sncRNA-1 was confirmed to be highly expressed, as quantified with the LNA^TM^ quantitative miRNA PCR system (Figure 3B).

The elevated expression of the mature sncRNA-1 in several independently isolated *Mtb* H37Rv 6230 clones enabled us to evaluate the impact of this sncRNA on *Mtb* H37Rv 6230 gene expression. Comparative RNA Seq with three independently isolated sncRNA-1 clones along with three vector controls revealed many differentially expressed genes (DEGs), selected based on a 1.5-fold cutoff (Figure 3C and Supplementary Table S2). A subset of these DEGs are shown in Figure 3D. KEGG GO term analysis was done on the DEGs using DAVID 6.8 (Huang et al., 2008; Huang Da et al., 2009). This analysis revealed several over- and under- represented pathways. Among these were elevated fatty acid metabolic processes coupled to acetyl-CoA, and diminished expression of transcripts coupled to catabolic processes involving aromatic compounds (Figure 3F). We analyzed the functions of the DEGs involved in fatty acid metabolism using Mycobrowser. This analysis revealed that many of the up-regulated genes were involved in fatty acid biosynthesis (Supplementary Table S3). Two such genes (starred in Figure 3D) were *desA2* (Rv1094), which catalyzes the conversion of saturated fatty acids to unsaturated fatty acids, and *fabG4* (Rv0242c), which is involved in the first reduction step of fatty acid biosynthesis. Using qRT-PCR, these two genes were confirmed to be upregulated in sncRNA-1 vs ctl vector clones (Figure 3E). Overall, these results suggested that sncRNA-1 positively regulates components of the fatty acid biogenesis pathway.

### sncRNA-1 Improves *Mtb* Growth in Oleic Acid Deficient Growth Media

**FIGURE 4:**
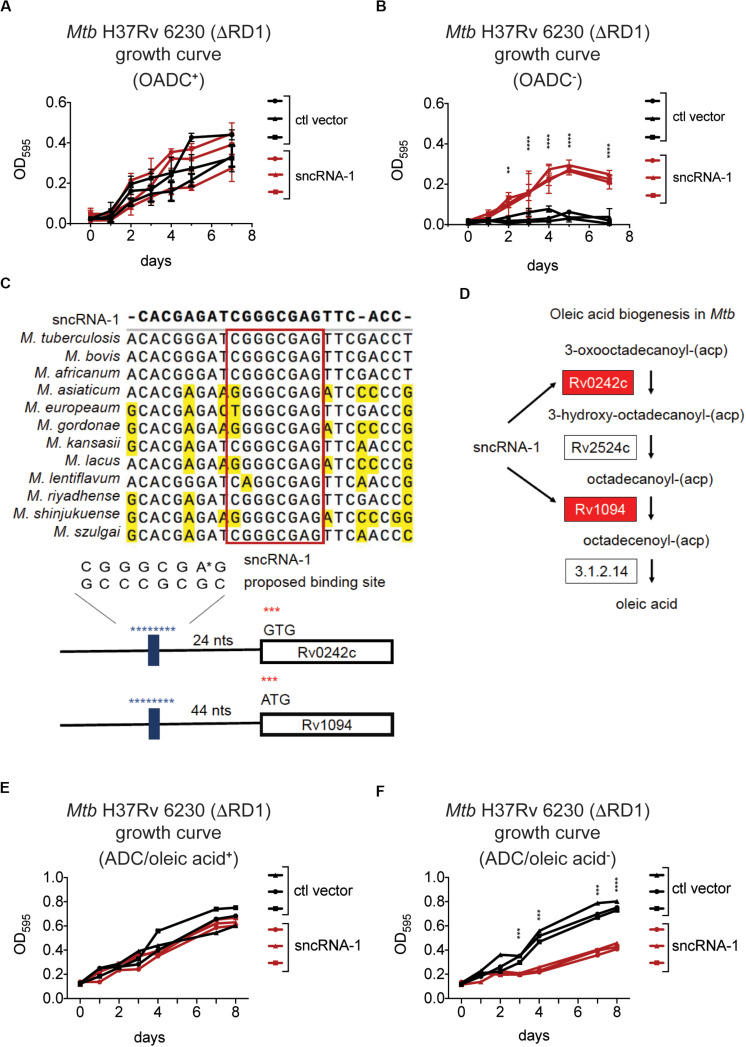
sncRNA-1 improves *Mtb* growth in oleic acid deficient growth media. **(A,B)** The fast growing *Mtb* H37Rv 6230 (lacking endogenous RD1 and sncRNA-1) clones expressing the control vector (black) or the sncRNA-1 vector (red) were grown in pan^+^ 7H9 media with OADC **(A)** or without OADC **(B)** for 7 days. The optical density (OD) at 595 nm was measured every 24 h. *p*-values are calculated using student *t*-test with paired two-tailed distribution (^∗∗^*p* < 0.01, ^****^p < 0.0001). **(C)** sncRNA-1 seed sequence predicted by homology analysis is shown. Proposed sncRNA-1 targets have sncRNA-1 binding site in their 5′ UTR, shown in blue. **(D)** The oleic acid biogenesis pathway is shown. This was modified from KEGG mycobacterial fatty acid biogenesis pathway available at: https://www.genome.jp/kegg-bin/show_pathway?map00061. Genes that were upregulated when sncRNA-1 was overexpressed in *Mtb* H37Rv 6230 are shown in red. **(E,F)** *Mtb* H37Rv 6230 clones expressing the control vector (black) or the sncRNA-1 vector (red) were grown in pan^+^ 7H9/ADC media with oleic acid **(E)** or without oleic acid **(F)** for 8 days. The optical density (OD) at 595 nm was measured every 24–48 h. *p*-values are calculated using student *t*-test with paired two-tailed distribution (^∗∗∗^*p* < 0.001, ^****^p < 0.0001).

The differential expression of genes involved in fatty acid biosynthesis in sncRNA-1 overexpressing clones suggested this sncRNA could confer a growth advantage to mycobacteria cultured in media lacking fatty acid rich supplements. To test this, multiple fast growing *Mtb* H37Rv 6230 clones, expressing either the control vector or the sncRNA-1 expression vector, were grown in rich media containing the fatty acid Oleic acid, along with Albumin, Dextrose, and Catalase (OADC^+^), or in media lacking OADC (OADC^–^). The *Mtb* H37Rv 6230 clones grown in OADC^+^ media had similar growth kinetics with/without sncRNA-1 (Figure 4A). However, the sncRNA-1 over-expressing clones were able to grow in OADC^–^, while those lacking sncRNA-1 failed to expand (Figure 4B). These data further support the interpretation that sncRNA-1 positively regulates *Mtb* growth in nutrient deficient media.

Small RNAs often regulate the expression of target transcripts by Watson–Crick base pairing through a 6–7 nts seed sequence (Gerrick et al., 2018; Girardin and McDonough, 2019; Mai et al., 2019). For this reason, a homology search was done comparing the sncRNA-1 nucleotide sequences in different mycobacterial species. This revealed a highly conserved 8-nt sequence (Figure 4C). Notably, the stem loop structure in which sncRNA-1 resides is also highly conserved (data not shown). The sRNA target prediction program TargetRNA2 was used with the putative 8-nt seed sequence of sncRNA-1 as a probe. Of the putative targets identified, two transcripts, Rv1094 (*desA2*) and Rv0242c (*fabG4*), had putative sncRNA-1 binding site in their 5’ UTRs (Figure 4C and Supplementary Table S4). Based on the KEGG mycobacterial fatty acid biogenesis pathway available at: https://www.genome.jp/kegg-bin/show_pathway?map00061, Rv1094 and Rv0242c are two of the four genes involved in the synthesis of octadecenoic acid (oleic acid) (Figure 4D and Supplementary Table S2). Both of these transcripts were upregulated in the sncRNA-1 overexpressing clones (Figure 3E). These findings suggest that sncRNA-1 targets *Mtb* genes involved in oleic acid biosynthesis. This would imply that the growth advantage of sncRNA-1 overexpressing clones in the OADC deficient media (OADC^–^) was primarily due to the induction of oleic acid synthesis genes. To test this, *Mtb* H37Rv 6230 clones expressing either the control vector or the sncRNA-1 expression vector, were grown in noncommercial 7H9/ADC^+^ media that was oleic acid sufficient (ADC/oleic acid^+^) or deficient (ADC/oleic acid^–^). Both *Mtb* H37Rv 6230 clones expressing ctl vector or sncRNA-1 vector had similar growth kinetics in regular media (Figure 4E). However, while the *Mtb* H37Rv 6230 clones expressing ctl vector had a growth deficit in the absence of oleic acid, the *Mtb* H37Rv 6230 clones expressing sncRNA-1 expanded without oleic acid supplementation (Figure 4F). The vector control clones did exhibit some growth, likely due the remaining ADC supplements providing some nutrients needed for mycobacterial growth (Figure 4F). Overall, the data suggested that sncRNA-1 positively regulates oleic acid production in *Mtb*, enabling mycobacterial growth in oleic acid-deficient media.

### sncRNA-1 Uses a Seed Sequence to Positively Regulate the Expression of Rv0242c Required for Oleic Acid Synthesis

**FIGURE 5:**
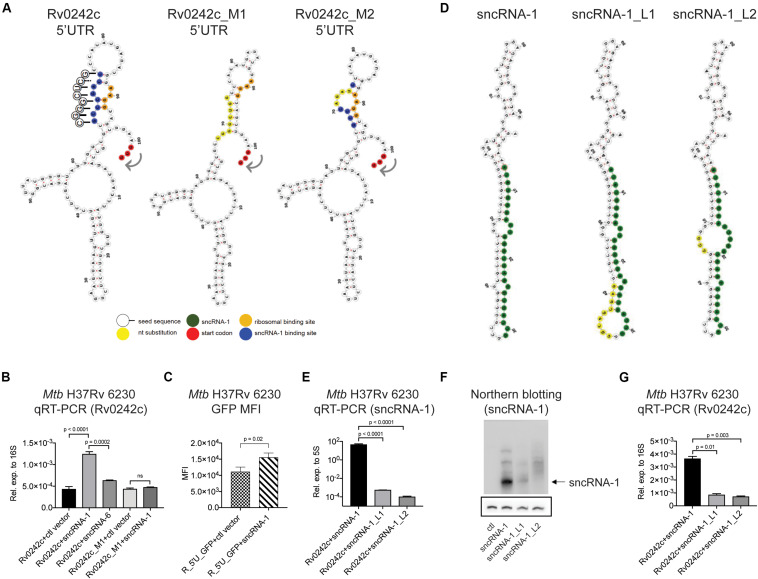
sncRNA-1 uses a seed sequence to positively regulate the expression of Rv0242c required for oleic acid synthesis. **(A)** Predicted secondary structure of the 5′ UTR of Rv0242c, Rv0242c_M1, and Rv0242c_M2 are shown, which were modeled using RNAfold web server. The start codon is shown in red, the proposed sncRNA-1 binding site is shown in blue, and mutated nucleotides are shown in yellow. The predicted ribosomal binding site, containing an AAGG sequence, hybridizes with the predicted sncRNA-1 binding site in the 5′ UTR of Rv0242c. The sncRNA-1 seed sequence interacting with the proposed sncRNA-1 binding site is shown in circles. The complementary sequences are shown with solid lines whereas the non-complementary sequence is shown with a dashed line. **(B)** Rv0242c along with its promoter region including the 5′ UTR was cloned into a protein expression vector called pSUM-Kan-MCS1-gfp. The kanamycin cassette was replaced by a Zeocin resistance cassette and GFP was replaced by the Rv0242c. Plasmids expressing the control vector or sncRNA-1 or sncRNA-6 were co-electroporated into *Mtb* H37Rv 6230 along with the Rv0242c expression vector. sncRNA-6 was used as a negative control. Individual transformants were obtained following selection in media containing two antibiotics. RNA was isolated from cultures at stationary phase, and Rv0242c expression was quantified relative to 16S rRNA by qRT-PCR. *p*-values are calculated using one-way ANOVA. **(C)** A reporter assay was developed in which the 5′ UTR of Rv0242c was cloned upstream of the cDNA for GFP. This construct was co-expressed with either ctl vector or the sncRNA-1 expression vector. Median GFP intensity was quantified in cultures at stationary phase. For statistics, student *t*-test was used. **(D)** Nucleotide substitutions were introduced in the hairpin structure of sncRNA-1 to disrupt its secondary structure and hence processing using site directed mutagenesis. Two distinct mutants were obtained. sncRNA-1 sequence is shown in green and the mutations are shown in yellow. **(E–G)** sncRNA-1, sncRNA-1_L1, or sncRNA-1_L2 was co-expressed with the putative target Rv0242c in *Mtb* H37Rv 6230. *p*-values are calculated using one-way ANOVA. **(E)** sncRNA-1 expression was quantified relative to 5S RNA as indicated in the axis labels. **(F)** sncRNA-1 expression was visualized with Northern blotting. The membrane was initially probed for sncRNA-1 and exposed for 24 h. sncRNA-1 was shown with an arrow. The same membrane was stripped and probed for 5S RNA for 3 h. 5S RNA was shown in the box. **(G)** Rv0242c expression was quantified relative to 16S RNA as indicated in the axis labels. *p*-values are calculated using one-way ANOVA.

To determine if sncRNA-1 directly regulated the expression of its targets, both the sncRNA and one of the putative target genes, Rv0242c, were expressed in *Mtb* H37Rv 6230. We expressed the target exogenously to ascertain the role of the 5′ regulatory elements of the gene. Experimentally, Rv0242c, along with its native promoter region, was cloned into a protein expression vector, pSUM-Kan-MCS1-gfp (Supplementary Figure S5A) (Eitson et al., 2012). The kanamycin cassette was replaced with a zeocin resistance cassette and GFP was replaced with Rv0242c. In combination with Rv0242c, three distinct RNA expression plasmids, either pKA-303 alone as a control vector, or containing sncRNA-1 or sncRNA-6, were electroporated into *Mtb* H37Rv 6230. sncRNA-6 was used as a negative control. Clones were isolated and grown in double selection media. The presence of sncRNA-1, but not control vector or sncRNA-6 caused a threefold increase in Rv0242c expression (Figure 5B, first three bars). This finding suggests that sncRNA-1 positively regulates Rv0242c expression by nucleotide-specific interactions with the 5′ UTR. To confirm this possibility, the putative sncRNA-1 binding site in the 5′ UTR of Rv0242c was mutated by site directed mutagenesis. Two distinct mutations were introduced and are referred to as Rv0242c_M1 and Rv0242c_M2 (Figure 5A). Rv0242c_M1 had all 8 nts in the putative sncRNA-1 binding site mutated. sncRNA-1 no longer potentiated the levels of Rv0242c, as the expression levels of the gene were similar to the control vector/Rv0242c_M1 combination (Figure 5B, last two bars). Rv0242c_M2 was generated with a 4-nts substitution in the proposed sncRNA-1 binding site (Figure 5A). Notably, the exogenous expression of Rv0242c was completely abrogated in the Rv0242c_M2 construct, regardless of whether the ctl vector or sncRNA-1 was co-expressed (Supplementary Figures S5B,C). This revealed a critical sequence in this sncRNA-1 target site needed for normal gene expression. Modeling the structure of the 5′ UTR of Rv0242c using a RNAfold structure prediction program revealed that the proposed sncRNA-1 binding site interacts near the predicted RBS (AAGG). Our data indicate this region is targeted by sncRNA-1, increasing the expression of Rv0242c. To further support this conclusion, a reporter assay was designed in which just the promoter region including the 5′ UTR of Rv0242c was cloned upstream of GFP (R_5′U_GFP). This was co-expressed with either the control vector or the sncRNA-1 expression vector in *Mtb* H37Rv 6230. sncRNA-1 enhanced the GFP intensity compared to the control vector, establishing that sncRNA-1 regulates the expression of the target through its 5′ UTR (Figure 5C).

To establish whether the mature sncRNA-1 was uniquely responsible for the targeting of Rv0242c, two distinct sets of nucleotide substitutions were introduced into the hairpin loop structure of the precursor form of sncRNA-1. We hypothesized that these mutations would affect the processing of sncRNA-1, which would change the targeting of Rv0242c. RNA secondary structure prediction programs suggested the two mutants, sncRNA-1_L1 and sncRNA-1_L2, would have larger loops that could impact sncRNA-1 formation (Figure 5D). sncRNA-1 expression was reduced at least 100-fold in the *Mtb* H37Rv 6230 clones expressing either sncRNA-1_L1 or sncRNA-1_L2 (Figure 5E). This was confirmed by Northern blotting showing a significant loss of the mature sncRNA-1 species when either mutant was over-expressed (Figure 5F). Plasmids containing these mutations were co-expressed with Rv0242c expression vector. Rv0242c expression was reduced 3–4-fold in either of the *Mtb* H37Rv 6230 clones expressing sncRNA-1 hairpin loop mutants compared to the intact sncRNA-1 sequence (Figure 5G). These findings illustrate that the functions of sncRNA-1 are dependent on the production of the mature 25-nt species. In addition, they further suggest the existence of a hairpin loop dependent sRNA processing system in *Mtb*.

### Locked Nucleic Acid Power Inhibitors Antagonize sncRNA-1 Functions in Intracellular Infections

**FIGURE 6:**
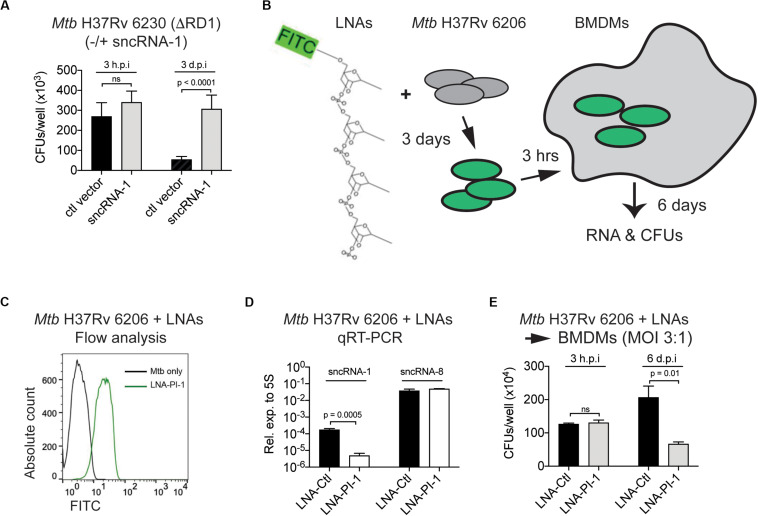
Locked nucleic acid power inhibitors antagonize sncRNA-1 functions in intracellular infections. **(A)** *Mtb* H37Rv 6230 expressing control vector or sncRNA-1 expression vector were used to infect BMDM. BMDM was lysed in 0.05% SDS 3 h or 3 days post infection. *Mtb* H37Rv 6230 was plated on 7H10 agar plates with hygromycin and colony forming units were counted 2–3 days after plating. **(B–E)** Loss of function assay was developed to knockdown sncRNA-1 expression in *Mtb* H37Rv 6206. **(B)** Experimental design is shown. Exponentially growing *Mtb* H37Rv 6206 was incubated with LNA-PI targeting sncRNA-1 (LNA-PI-1) for 3 days. **(C)** Cells were fixed in 2% PFA and fluorescence intensity was analyzed with flow cytometry. **(D)** BMDMs were infected with *Mtb* H37Rv 6206 that had been incubated with LNA-PI-1 or LNA-Ctl. After 6 days, RNA was extracted, and sncRNA-1 and sncRNA-8 expression quantified with miRCURY LNA^TM^ quantitative miRNA PCR system. Statistical significance was determined with student *t*-test. **(E)** Infected BMDMs were lysed in 0.05% SDS at 3 h and 6 days post infection. The remaining *Mtb* H37Rv 6206 mycobacteria were plated on 7H10 agar plates with hygromycin. CFUs were counted around 3 weeks after plating. Statistical significance was determined with one-way ANOVA.

Given the regulatory role of sncRNA-1 in regulating mycobacterial growth in nutrient depleted conditions through oleic acid production, we next examined how this sncRNA affected mycobacterial pathogenesis. *Mtb* H37Rv 6230 (ΔRD1, ΔpanCD) clones containing either a control vector or the sncRNA-1 expression vector was used to infect BMDM in pan^+^ media (MOI = 3:1). The CFU counts were quantified at 3 h and 3 days post infection. At 3 h post-infection, the number of CFUs/well was similar among all the clones tested. By 3 days post-infection, *Mtb* H37Rv 6230 clones expressing the control vector had reduced CFUs/well (Figure 6A), consistent with the fact that this mycobacterial auxotroph has a survival disadvantage without the RD1 locus (Guinn et al., 2004; Sambandamurthy et al., 2006). *Mtb* H37Rv 6230 overexpressing sncRNA-1 had a statistically significant 5–6-fold increase in CFUs/well, revealing a survival advantage (Figure 6A). The sncRNA overexpression assay was repeated with *Mtb* H37Rv 6206 (ΔpanCD, ΔleuC), which retains the RD1 region and is comparable to *Mtb* H37Rv in terms of the growth kinetics in leu^+^ pan^+^ media. BMDMs in leu^+^ pan^+^ media were infected with *Mtb* H37Rv 6206 overexpressing ctl vector or sncRNA-1 (MOI = 3:1) (Supplementary Figure S6A). The CFU counts were examined 6 days post infection as opposed to 3-days since the *Mtb* H37Rv 6206 auxotroph grows more slowly than the 6230 strain. The overexpression of sncRNA-1 increased the CFU/well count following *Mtb* H37Rv 6206 infections in BMDMs (Supplementary Figure S6B). Overall, these data show that sncRNA-1 overexpression supports *Mtb* H37Rv survival inside macrophages, which is revealed both in the presence and absence of the RD1 locus.

As a complementary approach to the aforementioned findings, a loss of function assay using LNA-PIs was undertaken. LNA-PIs are nuclease resistant, 14–16mer antisense RNAs that form high affinity duplexes with target miRNAs to disrupt the functions of a miRNA (Elmen et al., 2008; Fabani and Gait, 2008; Stein et al., 2010; Obad et al., 2011). These LNA-PIs have been successfully used to antagonize the function of miRNAs in eukaryotic cells without transfection requirements. Given the potential value for using such inhibitors in mycobacterial studies, a randomly designed LNA inhibitor that does not target any known miRNA was used as a negative control (LNA-Ctl). This control and one targeting sncRNA-1 (LNA-PI-1) were synthesized and tested in mycobacteria (Figure 6B). *Mtb* H37Rv 6206, which contains the endogenous sncRNA-1, was cultured with the LNA-PI-1 or LNA-Ctl. Flow cytometry analysis revealed that the LNA-PI-1 was readily incorporated in cultures of *Mtb* H37Rv 6206 without any transfection requirements (Figure 6C). *Mtb* H37Rv 6206 cultures grown in the presence of the LNA-Ctl or the LNA-PI-1 were used to infect macrophages. After 6 days of infection, sncRNA-1 was antagonized in the macrophages infected with *Mtb* H37Rv 6206 cultures containing the LNA-PI-1 compared to those with the LNA-Ctl (Figure 6D). The levels of an unrelated sncRNA, sncRNA-8, were unaffected by the LNA-PI-1, confirming the specificity of LNA-PI-1 against sncRNA-1 (Figure 6D). The functional impact of reducing sncRNA-1 expression on the survival of *Mtb* H37Rv 6206 in infected cells was assessed by CFU/well counts 3 hours and 6 days post infection. The cultures containing the LNA-PI-1 had a >2-fold decrease in CFU counts when compared to those containing the LNA-Ctl at the 6-day time point (Figure 6E). The LNA loss-of-function assay was repeated with the *Mtb* H37Rv 6230 clones overexpressing sncRNA-1 (*Mtb* H37Rv 6230 OE). *Mtb* H37Rv 6230 OE was incubated with LNA-PI-1 or LNA-Ctl (Supplementary Figures S6C,D). These LNA-treated mycobacterial clones were used to infect BMDM at MOIs of 3:1, and the CFUs/well determined 3-days post infection. In *Mtb* H37Rv 6230 overexpressing sncRNA-1, the CFUs/well decreased when sncRNA-1 was specifically targeted with the LNA (Supplementary Figure S6E). The CFU/well reduction in the presence of the LNA-PI-1 was not as dramatic in the *Mtb* H37Rv 6230 mycobacteria as that seen with the *Mtb* H37Rv 6206 auxotroph. This is likely because the *Mtb* H37Rv 6230 have a much faster doubling time, causing a dilution of the LNA power inhibitor as the mycobacteria expanded.

With the feasibility of antagonizing sncRNA-1 expression in mycobacteria, we addressed whether this would affect the expression of Rv0242c. We incubated the *Mtb* H37Rv 6230 clones overexpressing sncRNA-1 and Rv0242c with either the LNA-PI-1 or the LNA-Ctl. Following 18 h of incubation with the LNA-PIs, RNA was isolated and Rv0242c expression was quantified. This analysis revealed that when *Mtb* H37Rv 6230 OE was treated with LNA-power inhibitor targeting sncRNA-1 (LNA-PI-1), the levels of Rv0242c were reduced >2 fold, further supporting our conclusion that sncRNA-1 positively regulates Rv0242c (Supplementary Figure S5B, last two bars).

## Discussion

Many bacterial species, including mycobacteria, produce sRNAs under stress conditions (Arnvig and Young, 2009; Storz et al., 2011; Miotto et al., 2012; Haning et al., 2014; Gerrick et al., 2018). As most of these sRNAs have been identified with conventional RNA Seq data analysis procedures, those less than 50 nts in length are often overlooked. We applied an RNA sequencing strategy to capture the sRNAs <30 nts with *Mtb* H37Rv-infected macrophage cultures. Our interest in this size range stems from our earlier work on miRNAs (Belkaya et al., 2011; de la Morena et al., 2013; Hoover et al., 2016). Thirty-five distinct *Mtb* containing sncRNAs were detected in the infected macrophages. While many of these smaller RNAs could arise from larger transcripts that are degraded during intracellular infections, we focused our attention on three of the sncRNAs, sncRNA-1, sncRNA-6, and sncRNA-8 for the following reasons. These three sncRNAs had the highest sequence reads, their expression increased intracellularly over the course of an infection, and each had precursor sequences with the smallest MFEs that suggested stability.

We have focused our functional studies sncRNA-1 since it was not identified in any prior transcriptome studies and it exists within a key pathogenicity locus, RD1. RNA-seq of the fast-growing auxotroph mutant of *Mtb* H37Rv 6230 over-expressing sncRNA-1 suggested a novel role for this sncRNA in the positive regulation of two genes involved in oleic acid biogenesis, Rv1094 and Rv0242c. This was confirmed for Rv0242c, a gene that encodes FabG4, a non-canonical and essential 3-oxoacyl-thioester reductase (Gurvitz, 2009). Rv0242c has been implicated in mycobacterial resistance to streptomycin (Sharma et al., 2010). Regulation of Rv0242c by sncRNA-1 indicates a possible role for this sRNA in mycobacterial drug resistance. The production of oleic acid, an unsaturated fatty acid, also contributes to decreased membrane fluidity in the mycobacteria, improving their survival chances in harsh conditions such as the phagolysosome in macrophages (Bloch and Segal, 1956; Lee et al., 2013). Our findings suggest that sncRNA-1 regulates Rv0242c expression by direct sequence specific interactions involving a putative seed sequence in sncRNA-1, which is conserved among mycobacteria. This was supported by the mutagenesis of the 5′ UTR region of Rv0242c. The 5′ UTR region has a critical role in gene expression, which encompasses the RBS (Flentie et al., 2016). RNAfold was used to compare the predicted secondary structure of this region for the wild type Rv0242c along with the two mutants that we created (Figure 5A). A bending of the RBS was revealed that was unique to Rv0242c_M2, implying that the access of the translational machinery may be blocked, hence reducing Rv0242c_M2 stability and expression (Figure 5A and Supplementary Figure S5B). Overall, these data suggest that sncRNA-1 regulates the expression of Rv0242c through its 5′ UTR. Future studies will address the mechanism of regulation of Rv1094.

A role of sncRNA-1 in mycobacterial pathogenesis was supported by both gain- and loss- of function assays. sncRNA-1 was overexpressed in either *Mtb* H37Rv 6206 (ΔpanCD, ΔleuC) and *Mtb* H37Rv 6230 (ΔRD1, ΔpanCD), with the latter lacking endogenous sncRNA-1. Overexpression of sncRNA-1 significantly increased the number of CFUs/well in macrophages infected with *Mtb* H37Rv 6230 (3 days p.i.) compared to *Mtb* H37Rv 6206 (6 days p.i.). Such differences may be explained by the growth kinetics of the two mycobacterial strains. Six days corresponds to six division cycle in *Mtb* H37Rv 6206, whereas 3 days corresponds to 18 division cycle in *Mtb* H37Rv 6230. Therefore, the bacterial burden might have been determined when the phenotype would be more prominent for *Mtb* H37Rv 6230. Moreover, *Mtb* H37Rv 6230, developed as a part of vaccine development program, lacks the RD1 region important for *Mtb* pathogenicity (Sambandamurthy et al., 2006). Genes residing in this region include *esxA*, the gene product (ESAT-6) of which forms pores that span the membrane upon acidification of the phagolysosomes (Conrad et al., 2017). The function of these pores has been studied in the context of the secretion of virulence factors. However, it is possible that such pores are also used as channels to import substrates including fatty acids from the host. Therefore, in *Mtb* that had sncRNA-1 expression vector, lack of *esxA* may be rescued by sncRNA-1 overexpression followed by upregulation of fatty acid metabolism.

In infected cells, *Mtb* primarily relies on host lipids present in the macrophages, which are a rich source of oleic acid (Daniel et al., 2011; Maurya et al., 2019). While the upregulation of the *Mtb*-encoded genes that enable oleic acid synthesis might be counterintuitive, we speculate that sncRNA-1 upregulation at late stages of infection may be an anticipatory response to a potential upcoming fatty acid starvation. Macrophages restrict certain nutrients to starve bacteria upon infection (Cumming et al., 2018). However, the kinetics of oleic acid metabolism in *Mtb* infected macrophages remains incompletely understood up to date. Future studies will assess whether oleic acid used by macrophages modulates *Mtb* behavior in infected cells. Antagonizing sncRNA-1 using LNA-PI further supported a role for this sncRNA in mycobacterial survival. Specifically, antagonizing sncRNA-1 reduced the numbers of CFUs/well in macrophages. This suggests that sncRNA-1 may target additional genes that are capable of modulating mycobacterial infectivity. On a technical note, the LNA-PIs were readily taken up by both *Mtb* H37Rv 6206 and 6230 auxotroph strains and were capable of significantly reducing the levels of the sncRNAs in a sequence specific manner. The lack of any transfection requirements suggests that LNA-PIs will have wide applicability for diverse mycobacterial studies. Taken together, our data suggest that sncRNA-1 provides a pathogenic contribution to *Mtb* H37Rv, which is best revealed when the RD1 region is lacking.

The ability to overexpress sncRNA-1 with an RNA expression vector in *Mtb* H37Rv 6206 or 6230, independent of any eukaryotic infection model, confirm the existence of a prokaryotic RNA processing or degradation system for sRNAs. Moreover, abrogation of the sncRNA-1 processing in sncRNA-1 mutants suggests that sncRNA-1 processing is structure dependent. This was in agreement with a comparison of the levels of the sncRNA-1, wherein the 25-nt mature form, was only detected at high levels in *Mtb* expressing the wild type construct but not the mutant constructs (Figure 5). To investigate if this processing was conserved, we repeated a site directed mutagenesis analysis for sncRNA-6. This was done in *M. avium* using the pKA-303 over-expression system (Supplementary Figure S7). Similar to sncRNA-1, two distinct mutations were introduced in the hairpin loop structure, with one at the presumed cleavage site (Supplementary Figure S7). sncRNA-6 was no longer processed into the mature form when as few as 3 nts in the putative cleavage bulge were mutated. In addition, disrupting the secondary structure of sncRNA-6 by mutagenesis abrogated its processing, leading to the presence of a larger RNA transcript (Supplementary Figure S7). In most bacteria, the interactions between sRNAs and their targets are mediated by a widely conserved chaperone called Hfq (Cech et al., 2016). Interestingly, *Mtb* has no Hfq homolog and the sRNA–mRNA interactions are thought to be mediated by high GC content (65%) of *Mtb* genome (Arnvig, 2019). However, the sncRNAs identified in our screen have about 50% GC, implying the existence of other RNA binding/processing proteins in *Mtb*. While these experiments support a processing system that is sequence and hairpin loop dependent, we cannot rule out the possibility that many of the other 35 *Mtb*-encoded sncRNAs are from degradation processes. Future studies will focus on identifying the putative RNA binding and/or processing proteins required for generating sncRNA-1 and sncRNA-6.

In conclusion, we report on the identification and characterization of a group of *Mtb*-encoded sRNAs, termed sncRNAs, which are produced by pathogenic strains of *Mtb* in infected eukaryotic cells. One such sncRNA, sncRNA-1 supported mycobacterial growth, partly via the positive regulation of enzymes coupled to oleic acid synthesis. It will be informative to identify the mechanism of sncRNA biogenesis, as this may provide a new target for modulating mycobacterial infectivity.

## Data Availability Statement

The original contributions presented in this study are publicly available on NCBI under accession number GSE146228. This data can be found at: https://www.ncbi.nlm.nih.gov/geo/query/acc.cgi?acc=GSE146228.

## Ethics Statement

The animal study was reviewed and approved by Mouse animal work described in this manuscript has been approved and conducted under the oversight of the UT Southwestern Institutional Animal Care and Use Committee (APN number 2015-101247). Mice were housed in a specific pathogen-free facility at UT Southwestern Medical Center.

## Author Contributions

FC, SS, SB, and NO performed the experiments. FC, SS, PR, ID, SB, MC, EW, DK, TG, and NO analyzed the data. FC and NO prepared the figures and tables. FC, SS, ID, and NO performed statistical analyses. FC and NO wrote the manuscript. All authors contributed to the article and approved the submitted version.

## Conflict of Interest

The authors declare that the research was conducted in the absence of any commercial or financial relationships that could be construed as a potential conflict of interest.

## Footnotes

1 http://rna.tbi.univie.ac.at/cgi-bin/RNAWebSuite/RNAfold.cgi

2 http://nebuilder.neb.com/#!/

3 https://david.ncifcrf.gov/
