# Supplementary material for: sncRNA-1 Is a Small Noncoding RNA Produced by Mycobacterium tuberculosis in Infected Cells That Positively Regulates Genes Coupled to Oleic Acid Biosynthesis
Source: Front Microbiol. 2020 Jul 28;11:1631. doi: 10.3389/fmicb.2020.01631 (PMC7399025; doi:10.3389/fmicb.2020.01631)
Supplement: Supplementary file 1 [file Data_Sheet_1.pdf]

Supplementary Material

Supplemental Figure Legends

Figure S1. **Small RNA screening strategy in *Mtb*-infected THP-1 macrophages undertaken by LC Sciences.** miRNA sequencing data report for the 9 samples; 3 each for THP-1 cells independent of an infection and THP-1 cultures infected with *Mtb* H37Rv for 3 days and 6 days. LC Sciences provided the data.

Figure S2. **Time course of *hsa*-miRNA expression profile in *Mtb* infected THP-1 macrophages.** Human miRNAs are differentially modulated over time in *M. tuberculosis*-infected macrophages. THP-1 macrophages were infected with *Mtb* H37Rv (MOI = 10:1). Prior to infection and on days 3 and 6 post-infection, RNA was extracted, and small RNAs sequenced, and mapped to *hsa*-miRNAs. The number of sequence-reads for 48 of the most highly expressed human miRNAs are shown, using triplicate samples/group. Error bars are indicated. Statistical significance was indicated by one (*, $p < 0.05$), two (**, $p < 0.005$), or three asterisks (***, $p < 0.0005$).

Figure S3. Northern blotting for *Mtb* 5S RNA in *Mtb* H37Rv 6206 infected BMDM over the course of infection. BMDM were infected with *Mtb* H37Rv 6206 (MOI = 10:1). RNA was isolated 3 hours- 3 days-, or 6 days-post infection. Samples were run on polyacrylamide gel and transferred to a Nylon N+ membrane. Membrane was probed with probes specific for *Mtb* 5S RNA and exposed overnight.

Figure S4. pKA-303 plasmid map for sncRNA-1 expression vector. The RNA expression vector pKA-303 which was used to express sncRNA-1 in *Mtb* H37Rv 6230 (Δ*RD1*, Δ*panCD*) is shown. Kanamycin resistance cassette was replaced with hygromycin.

Figure S5. **Expression levels of Rv0242c and sncRNA-1 in *Mtb* co-expressing wild-type and mutated versions of the 5' UTR of Rv0242c in the presence/absence of sncRNA-1.** (A) The plasmid used to express Rv0242c along with its 5' UTR is shown. (B) Expression of Rv0242c was quantified in *Mtb* H37Rv 6230 expressing Rv0242c or Rv0242c_M2 and ctl vector or sncRNA-1. In the last two bars, Rv0242c was quantified in *Mtb* H37Rv 6230 overexpressing sncRNA-1 treated with LNA Ctl or LNA-PI-1. (C) Expression of sncRNA-1 was quantified in *Mtb* H37Rv 6230 expressing Rv0242c or Rv0242c_M1 or Rv0242c_M2 and ctl vector or sncRNA-1 vector.

Figure S6. Infection assays with sncRNA-1 overexpressing clones of *Mtb* H37Rv 6206 and sncRNA-1 knock down in *Mtb* H37Rv 6230. (A) sncRNA-1 expression vector was electroporated to *Mtb* H37Rv 6206 and colonies were selected on plates with hygromycin. Clones were grown in culture and RNA was isolated. sncRNA-1 was quantified to confirm overexpression. (B) *Mtb* H37Rv 6206 clones overexpressing sncRNA-1 were used to infect BMDM. BMDM was lysed in 0.05% SDS 3 hours or 6 days post infection. *Mtb* was plated on 7H10 agar plates with hygromycin and colony forming units were counted 3 weeks after plating. (C) *Mtb* H37Rv 6230 expressing sncRNA-1 expression vector was incubated with LNA-Ctl or LNA-PI-1 overnight. Cell were fixed in 2% PFA and fluorescence was assessed using flow cytometry. (D) sncRNA-1 and sncRNA-8 was quantified with miRCURY LNA^{TM} quantitative miRNA PCR system in *Mtb* H37Rv 6230 overexpressing sncRNA-1 treated with LNA-Ctl or LNA-PI-1 overnight. (E) These cells were used to infect BMDM.

BMDM was lysed in 0.05% SDS 3 hours or 3 days post infection. *Mtb* was plated on 7H10 agar plates with hygromycin and colony forming units were counted 2 days after plating.

Figure S7. Site directed mutagenesis of sncRNA-6. Nucleotide substitutions were introduced in the hairpin structure of sncRNA-6 to disrupt its secondary structure and hence processing using site directed mutagenesis similar to sncRNA-1. The plasmids were expressed in *Mycobacterium avium*. (A) Two distinct mutants were obtained named as sncRNA6_L1 and sncRNA-6_L2. sncRNA-6 sequence is shown in blue and the mutations are shown in red. (B) SncRNA-6 expression was visualized with Northern blotting. The membrane was initially probed for sncRNA-6 and exposed for 24 hours. SncRNA-6 was shown with an arrow. The same membrane was stripped and probed for 5S RNA for 3 hours. 5S RNA is shown as labeled.

Figure S1. Small RNA screening strategy in *Mtb*-infected THP-1 macrophages undertaken by LC Sciences

I. PROJECT INFORMATION

Table 1. Sample, service and project tracking information

**A: Project information**	
Customer Sample Name	RV-THP_D6-3
Sample Species	*Homo sapiens*
Sample Received Date	07/13/2012
Service Requested	microRNA Discovery Sequencing Service
LCS Project Number	5015
LCS Sample ID	RV-THP_D6-3

**B: Database information**		
Reference or Database Sequences	WEBlink and Information	Version or Built Date
miRNA(miRs) database	ftp://mirbase.org/pub/mirbase/CURRENT/; Specific species: hsa; Selected species: ptr, ppy, ppa, ggo, ssy, mml, mne, pbi, age, lla, sla, lca, mmu, rno, cgr, bta, oar, eca, oan, cfa, ssc, mdo, meu, sha	v19.0
Pre-miRNA(mirs) database	ftp://mirbase.org/pub/mirbase/CURRENT/; Specific species: hsa; Selected species: ptr, ppy, ppa, ggo, ssy, mml, mne, pbi, age, lla, sla, lca, mmu, rno, cgr, bta, oar, eca, oan, cfa, ssc, mdo, meu, sha	v19.0
Genome database	ftp://ftp.ncbi.nih.gov/genomes/H_sapiens/	37.1
mRNA database	ftp://ftp.ncbi.nih.gov/genomes/H_sapiens/RNA/	37.1
Customer database	NA	NA

2575 W. Bellfort, Suite 270, Houston, Texas 77054
Tel. (713)664-7087, Fax (713)664-8181

II. DATA REPORT

A. Terminologies Used

Table 2. Terminologies used in data analysis

Term	Description
Copy Number or Count	Number of sequ seqs in the same unique seq family
Mapping	Blasting a sequence to a reference database
miRBase	A searchable database of published miRNA sequences and annotation; http://mirbase.org
mir	Pre-miRNA registered in miRBase
miR	Mature miRNAs registered in miRBase
RepBase	Prototypic sequences representing repetitive DNA from different eukaryotic species; http://www.girinst.org/repbase
RFam	Collection of many common non-coding RNA families except micro RNA; http://rfam.janelia.org
Reads	DNA sequences from reading of sequencing intruments
Sequ Seq or Reads	Raw sequencing reads generated in after image extraction and base-calling
Unique Seq	Family of sequ seq with same sequence
Selected species	A combination of species defined by user
Specific species	Species of the sample analyzed

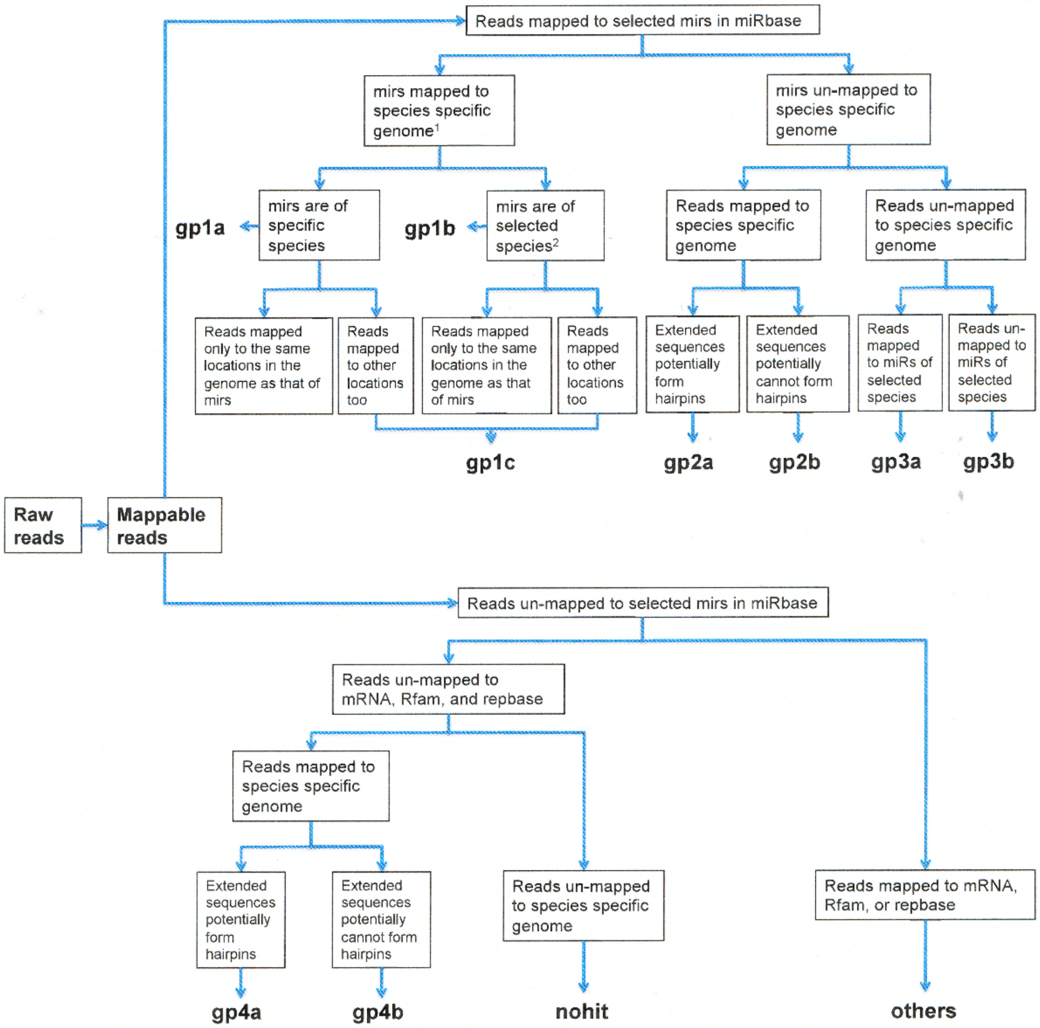

Figure 2. Data analysis flowchart

[1] *Homo sapiens*
[2] Mammalia

B. Length Distribution of Mappable Reads

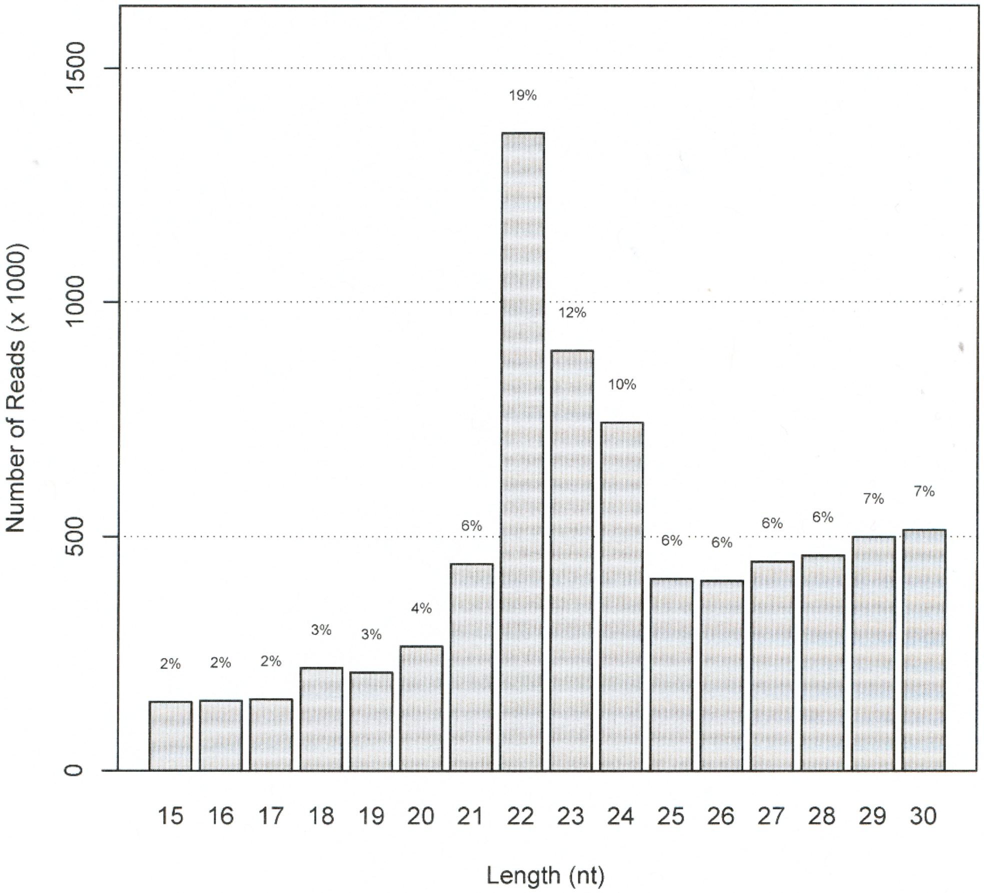

Figure 3. Length distribution of mappable reads

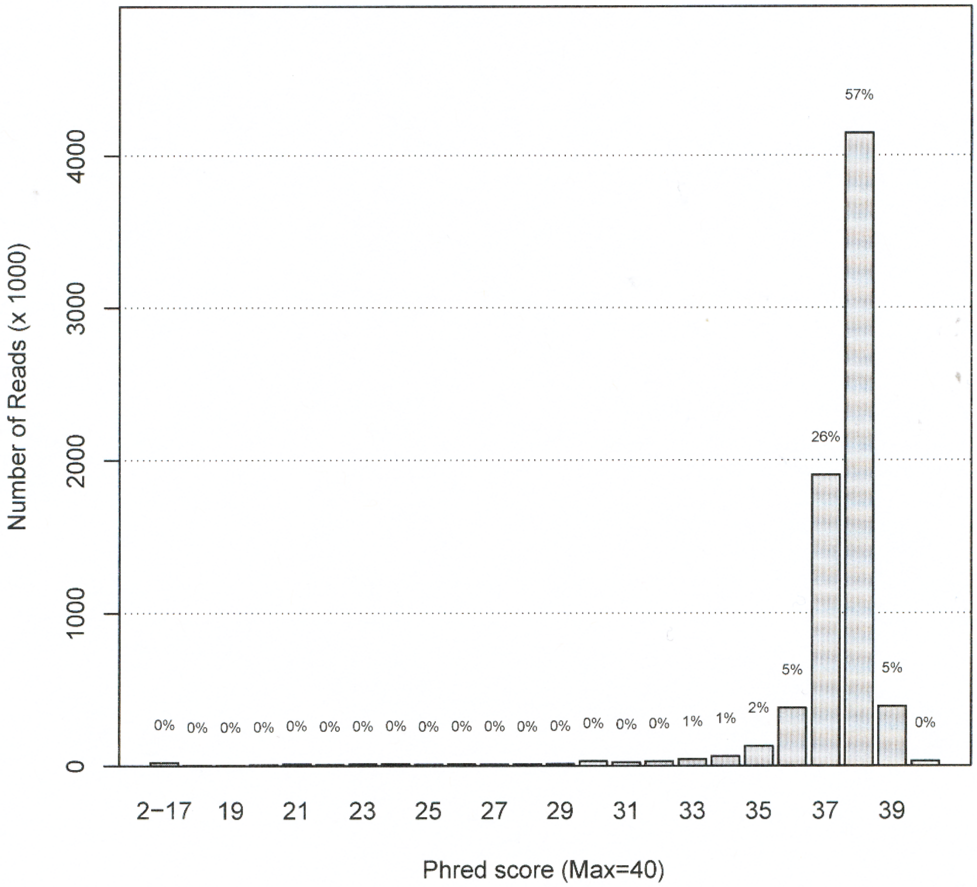

Figure 4. Histogram of the average phred score[1] per base of mappable reads

[1] Phred score larger than 30 stands for probability of incorrect base calls less than 1 in 1,000 (above 99.9% accuracy) in one sequencing read.

2575 W. Bellfort, Suite 270, Houston, Texas 77054
Tel. (713)664-7087, Fax (713)664-8181

Figure S2. Time course of *hsa*-miRNA expression profile in *Mtb* infected THP-1 macrophages

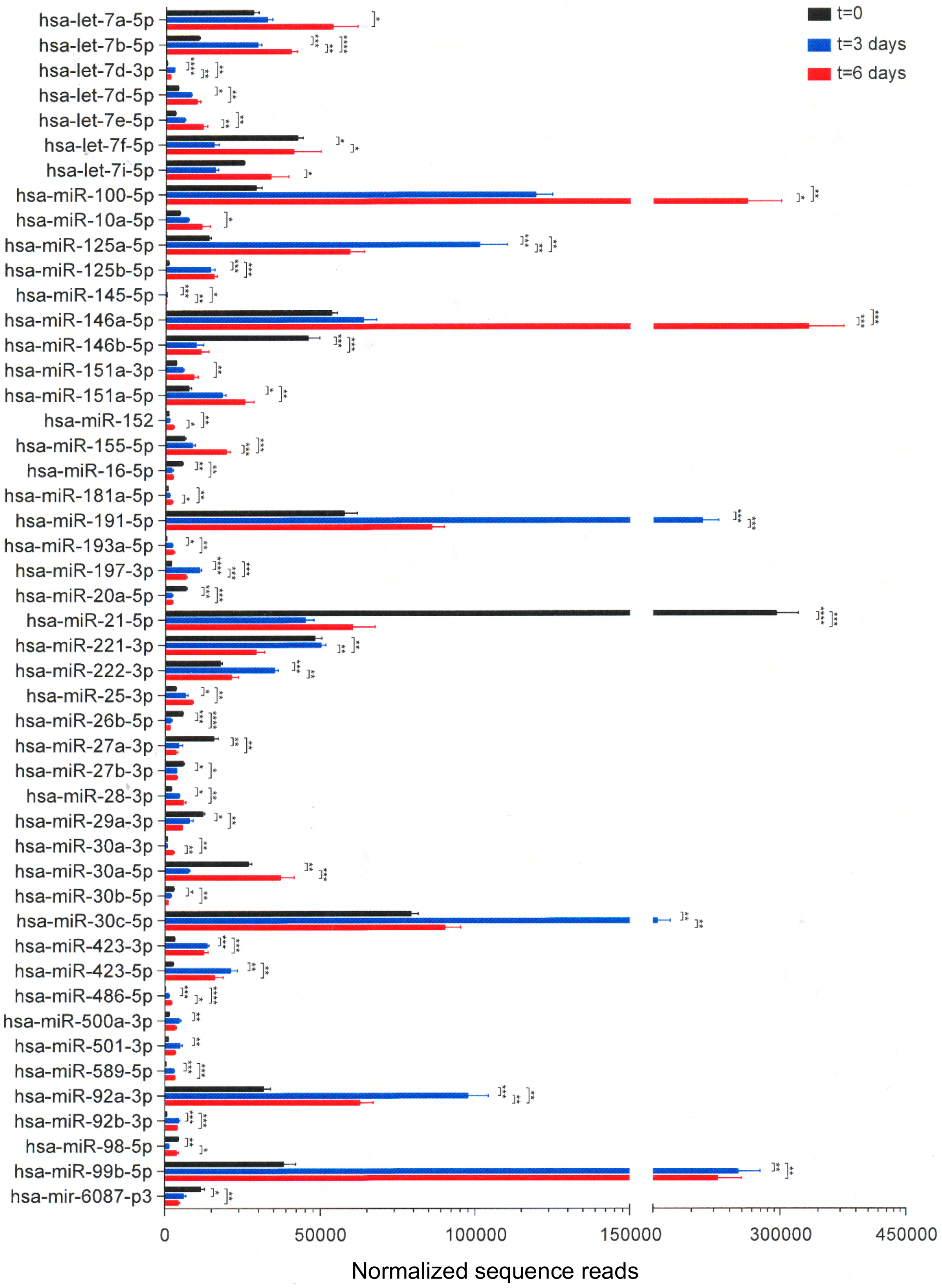

Figure S3. Northern blotting for *Mtb* 5S RNA in *Mtb* H37Rv 6206 infected BMDM over the course of infection

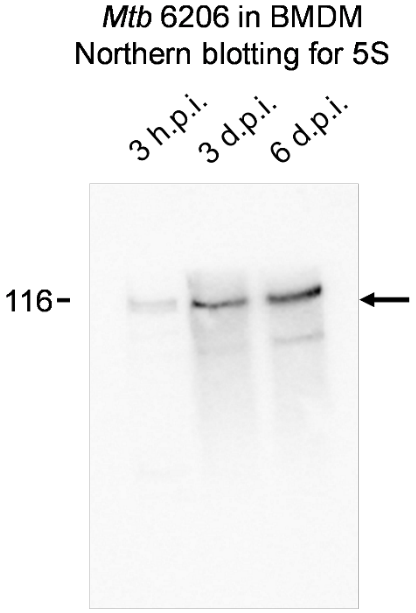

Figure S4. pKA-303 plasmid map for sncRNA-1 expression vector

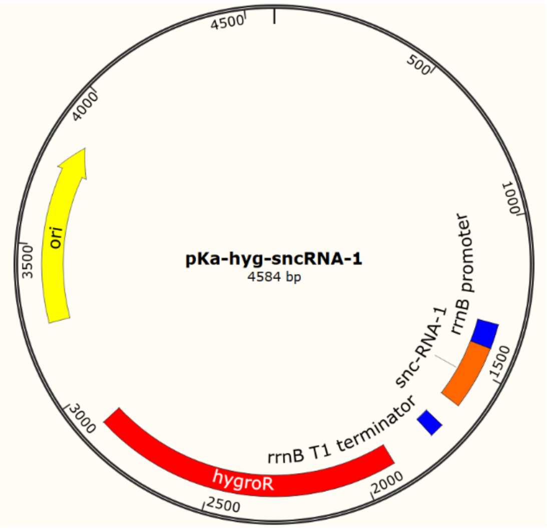

Figure S5. Expression levels of Rv0242c and sncRNA-1 in *Mtb* co-expressing wild-type and mutated versions of the 5' UTR of Rv0242c in the presence/absence of sncRNA-1

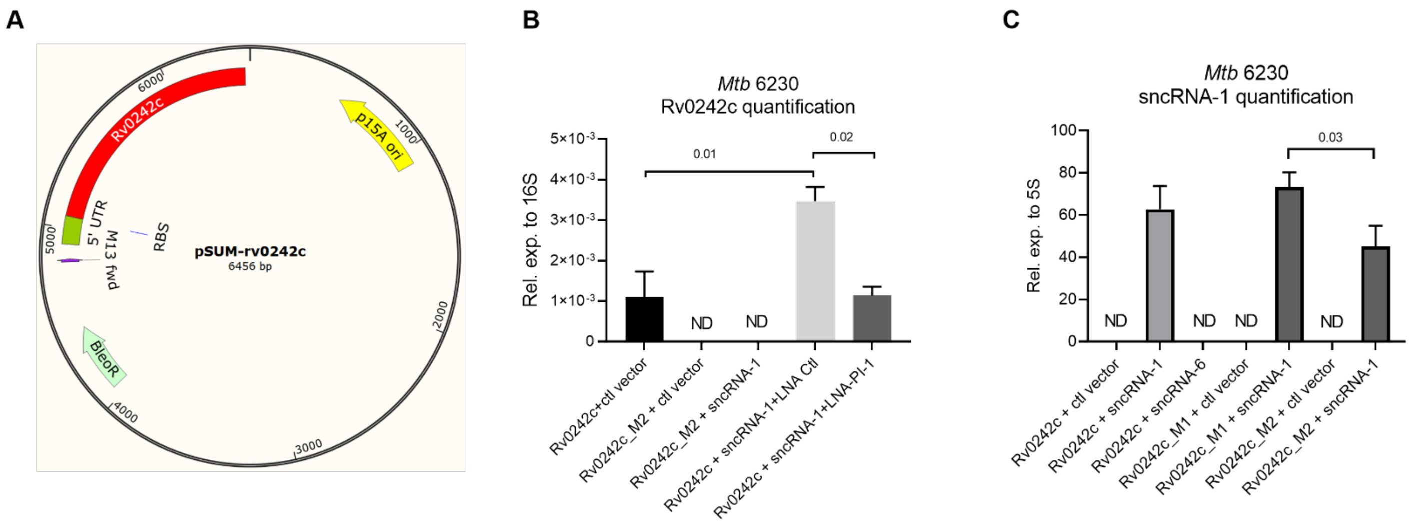

Figure S6. Infection assays with sncRNA-1 overexpressing clones of *Mtb* H37Rv 6206 and sncRNA-1 knock down in *Mtb* H37Rv 6230

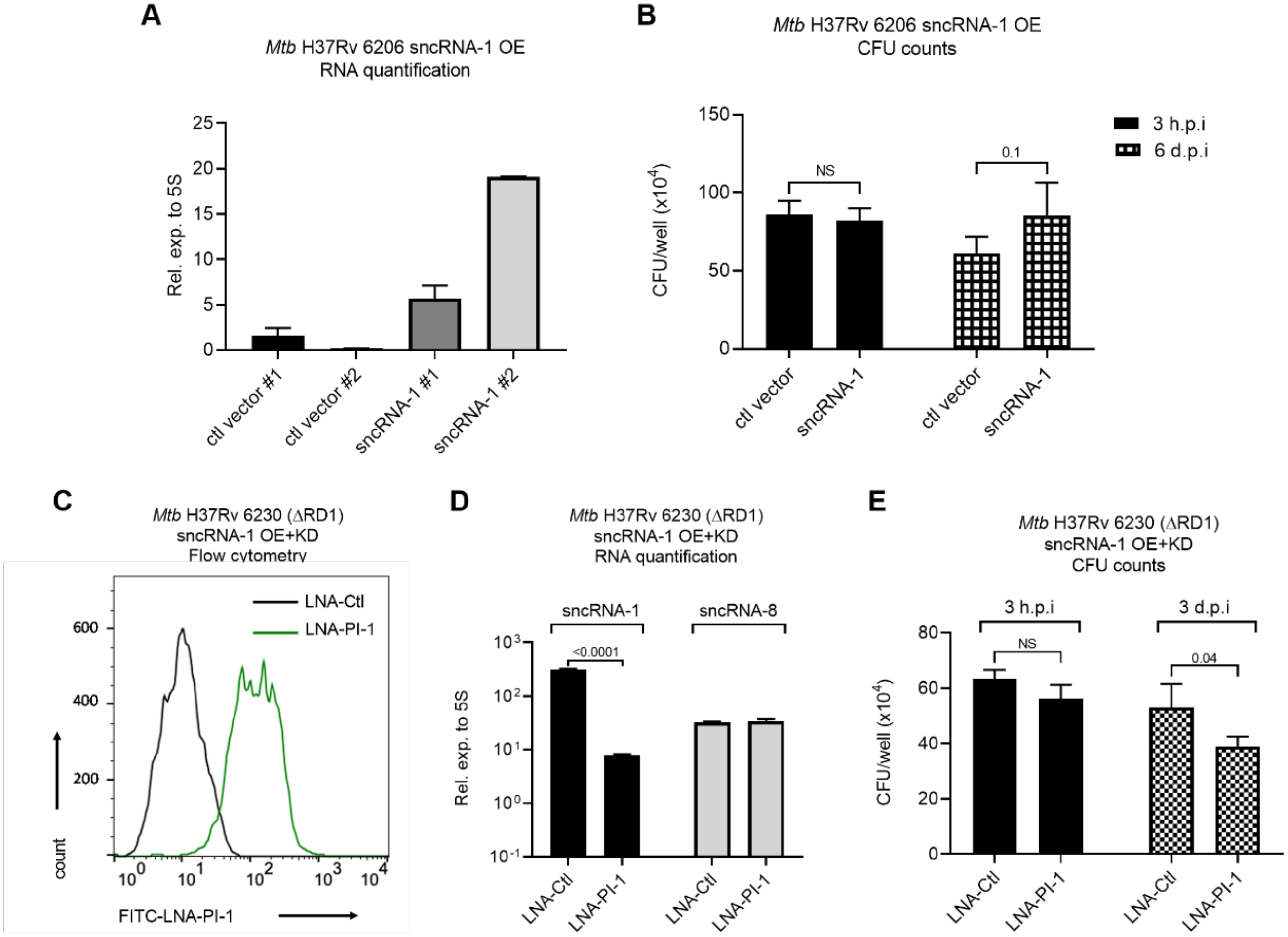

Figure S7. Site directed mutagenesis of sncRNA-6

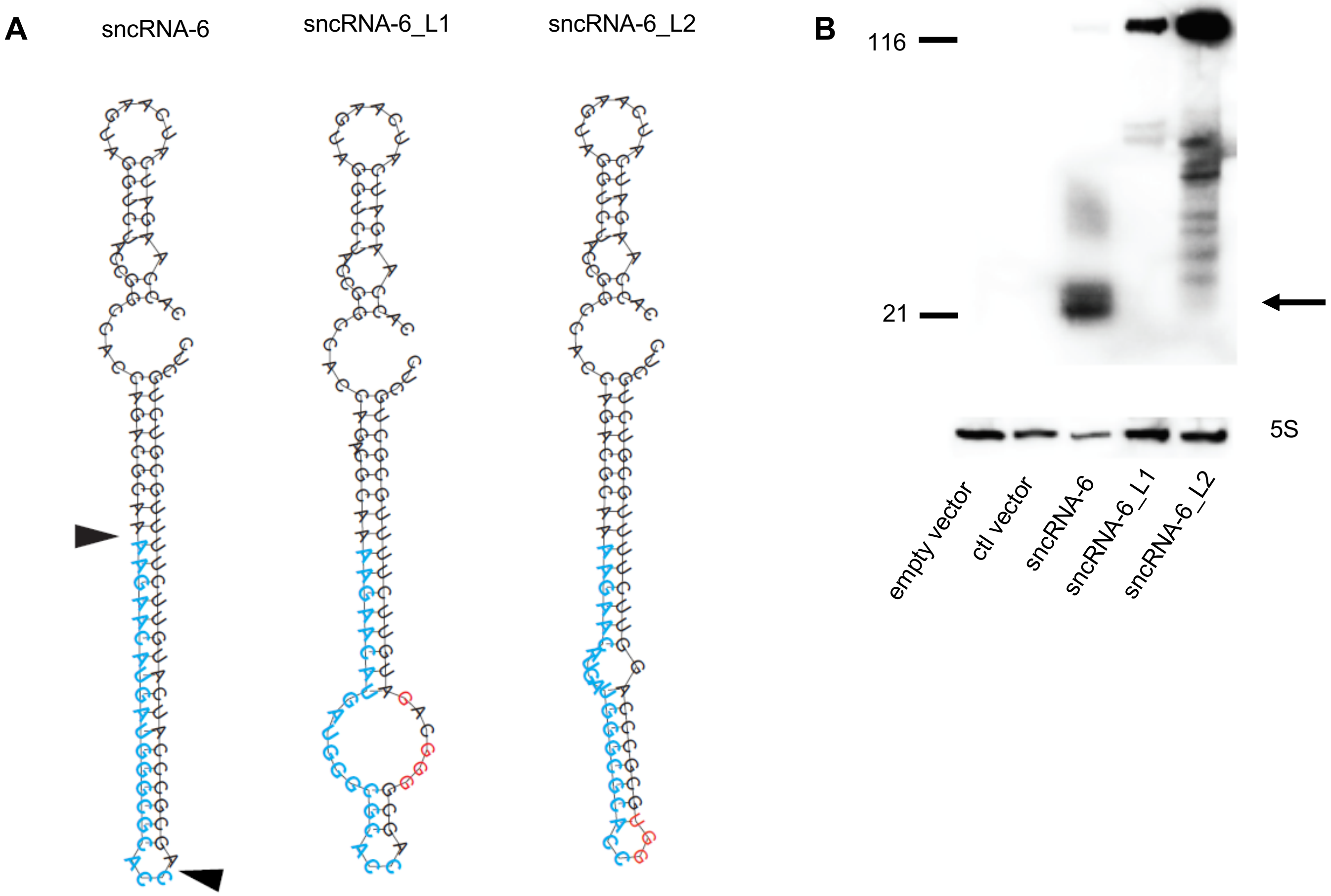

Table S1. Identification and genomic location of 35 distinct *M. tuberculosis*-encoded small noncoding RNAs produced in infected human macrophages

sncRNA designation[a]	Nucleotide Sequence	Length	Genomic Location[b]	Nearby coding sequence[c]	Other designations
sncRNA-1[d]	ACACGGGAUCGGGCGAGUUCGACCU	25	4352927-4352951	b/w[f] esxA (Rv3875) and Rv3876	ncRv13875A
sncRNA-2	AAGACACACGGAUACCCUUUGC	22	3343151-3343172	b/w mutT (Rv2985) and hupB (Rv2986c)	ncRv12985c
sncRNA-3	ACGGGCAGACUAGAGUACUGCAGGGGAGAC	30	1472478-1472507	rrs (Rvnr01)	ncRv0001
sncRNA-4[g]	GAAAUGACGCAAUGACCUCU[e]	20	3481440-3481459	Rv1199c and b/w Rv1200	ncRv11199A
sncRNA-5[d,g]	CGUUUCGAAGGAUCACGCGAUGACCGCCC[e]	29	3481390-3481418	b/w Rv1199c and Rv1200	ncRv11199B
sncRNA-6[g]	AAAGAACAUGAUGGGCGCACC[e]	21	786003-786083	b/w iron elongation factor (Rv0685) and (Rv0686)	ncRv10685
sncRNA-7	UGGUCAGAGAUGCAGUAACUC	21	4071678-4071698	b/w Rv3632 and Rv3633	ncRva13632c
sncRNA-8[d]	ACUCAAUAGUGUGUUUGGUGGUUU[e]	24	1471701-1471724	b/w murA (Rv1315) and rrs (Rvnr01)	ncRv11315 (mcr3)
sncRNA-9	CGGCCGGUUCAUAUGACAC[e]	19	1518217-1518237	b/w fabG (Rv1350) and Rv1351	ncRv11350
sncRNA-10	AAAGGAGGUGACUCGAUGCU	20	51723-51742	b/w Rv0047c and b/w Rv0048c	ncRv10047
sncRNA-11	GAUCCCGAAGAUUCGCUCC[e]	19	675996-676016	b/w PE_PGRS7 (Rv0578c) and Rv0579	ncRv10578
sncRNA-12	CUACCGGCCACCAGACGCAAAAGA	24	786014-786037	b/w tuf (Rv0685) and Rv0686	ncRv10685
sncRNA-13	CCGAGUCCUGAGGGCUGCAGUGACCCC	27	2096781-2096807	b/w Rv1846c and Rv1847	ncRv11846
sncRNA-14	CAACCGCGAAAUGACACUGAUGU	23	3640359-3640381	b/w whiB2 (Rv3260c) and fbiA (Rv3261)	ncRv13260
sncRNA-15	GCGUGAGUCGUUAAAGGUUGAUC[e]	23	3621447-3621469	b/w Rv3241c and Rv3242c	ncRv13241
sncRNA-16	CAAGCGUGUUGUUUGAGAACU	21	1471683-1471703	b/w murA (Rv1315) and rrs (Rvnr01)	ncRv11315
sncRNA-17	CGAAAUCUUGUUUGACGCGGAC[e]	22	1547634-1547655	b/w Rv1374c and Rv1375	ncRv11374 (MTS1082)
sncRNA-18	UCAGGGUCGGACAUAUCGCCUCC	23	4071702-4071724	b/w Rv3632 and Rv3633	ncRv13632c
sncRNA-19	AGAGUCACGCCGGGUCUGCCC	21	1413108-1413128	b/w Rv1264 and Rv1265	ncRv11264c (mcr11)
sncRNA-20	UGUCUGCUCGCCGAGGCCUACC[e]	22	456251-456272	b/w secE2 (Rv0379) and into Rv0380c	ncRv10379
sncRNA-21	CUUGCCCCAUCGUUCGUUGACUCUGCGUC	29	293713-293741	b/w fadA2 (Rv0243) and fadE5 (Rv0244c)	ncRv10243 (mcr14)
sncRNA-22	CCAGUGAUCGGCGGUCUC[e]	18	2626602-2626624	b/w PPE18 (Rv1196) and esxK (Rv1197)	ncRv11196
sncRNA-23	CGUGGUGGACGCAGAGUCAACGGA	24	3226334-3226357	b/w ffh (Rv2916c) and Rv2917	ncRv12916
sncRNA-24	UGAGUGGUCGGUAGUUGUC	19	4303389-4303411	b/w Rv2979c and Rv2980	ncRv12979
sncRNA-25	AACGAUCAGUAGUAGGCC	18	1196209-1196230	b/w echA9 (Rv1071c) and Rv1072	ncRv11071
sncRNA-26[g]	CGGCAACUGAAUACUGACC	19	4075666-4075684	b/w Rv3190c and Rv3191c	ncRv13190
sncRNA-27	CGCAACCUGGCCACCAAUUC	20	4318492-4318515	b/w PPE55 (Rv3347c) and Rv3348	ncRv13347
sncRNA-28	CGUUUGAGCGCACUCUGAGAGGU	23	4352983-4353005	b/w esxA (Rv3875) and Rv3876	ncRv13875B
sncRNA-29	UUUGGAGGUUCUGAGAUGAGUCC	23	611046-611069	b/w Rv0518 and Rv0519c	ncRv10518c
sncRNA-30	GAGCCGUCGGGACCACACAGUCAUU[e]	25	3862549-3862573	b/w rplM (Rv3443c) and esxT (Rv3444c)	ncRv13443
sncRNA-31	AGAUUGUUCAUCGGGGGCU[e]	19	892259-892277	b/w cfp29 (Rv0798c) and Rv0799c	ncRv10798

sncRNA-32	CGGUAGCCAGUAGUUCAUCC	20	333375-333394	b/w Rv0277c and PE_PGRS3 (Rv0278c)	ncRv10277
sncRNA-33	UGGUUUGUCGCGUUGUUCGU	20	1472422-1472441	rrs (Rvrn01)	ncRv0001B
sncRNA-34	CAAGAUGUCGAUUGCUCACC	20	3621389-3621408	b/w Rv3241c and Rv3242c	ncRv13241
sncRNA-35	CCUAGUUAUCUGCGCCGAGCGUGAAC	26	3987361-3987386	b/w Rv3547 into Rv3548c	ncRv13547c

[a]sncRNA number determined by p-value significance, calculated from n=3/time point
[b]Location on the *Mtb* H37Rv genomic sequence
[c]Sequence on or near coding sequences
[d]sncRNAs (sncRNA-1, sncRNA-5, sncRNA-8, sncRNA-19, sncRNA-26) detected in TB infected human and/or monkey lung tissue
[e]Sequences independently detected in *Mtb*-infected macrophages with an initial pilot small RNA discovery assay that was performed
[f]b/w = between the indicated coding proteins
[g]implicated in multiple locations

Table S2. Differentially expressed genes in *Mtb* H37Rv 6230 overexpressing sncRNA-1

down in sncRNA-1 OE			up in sncRNA-1 OE	
Rv0450c	Rv0826		MTB000034	Rv1311
Rv1162	Rv2050		MTB000049	Rv1448c
Rv0769	Rv2130c		MTB000039	Rv1599
Rv1863c	Rv2146c		Rv1095	Rv1653
Rv0011c	Rv2316		MTB000004	Rv1821
Rv0230c	Rv2374c		MTB000011	Rv1908c*
Rv0047c	Rv2388c		MTB000005	Rv1932
Rv1129c	Rv2413c		MTB000046	Rv1996
Rv1353c	Rv2466c		Rv0242c*	Rv2031c
Rv1461	Rv2643		Rv0665	Rv2210c
Rv0009	Rv2687c		MT18B_0033	Rv2247
Rv1082	Rv2699c		MTB000044	Rv2362c
Rv0780	Rv2706c		MTB000040	Rv2418c
Rv1467c	Rv3052c		Rv0694	Rv2426c
Rv1132	Rv3053c		MTB000047	Rv2538c
Rv1473	Rv3157		Rv1063c	Rv2725c
Rv0374c	Rv3198A		MTB000025	Rv2782c
Rv0049	Rv3207c		MTB000006	Rv2783c
Rv1852	Rv3221c		Rv1094*	Rv2839c
Rv0359	Rv3223c		MTB000024	Rv2878c
Rv1221	Rv3277		MTB000050	Rv2902c
Rv1472	Rv3462c		Rv0905	Rv2907c
Rv1334	Rv3464		Rv0241c	Rv2941
Rv1304	Rv3465		Rv0191	Rv3118
Rv0680c	Rv3516		MTB000035	Rv3133c
Rv1222	Rv3526		Rv0020c	Rv3140
Rv0383c	Rv3531c		MTB000029	Rv3285
Rv0269c	Rv3534c		MTB000021	Rv3359
Rv0249c	Rv3535c		MTB000015	Rv3777
Rv1894c	Rv3536c		Rv1157c	
Rv0767c	Rv3543c		MTB000038	
Rv0197	Rv3553		MTB000009	
Rv0821c	Rv3569c		MTB000041	
Rv1298	Rv3574		Rv0187	
Rv1292	Rv3626c		Rv0157	
Rv0764c	Rv3628		MTB000036	
Rv0870c	Rv3634c		MTB000043	
Rv1428c	Rv3684		Rv0243	
Rv3818	Rv3809c		MTB000042	
Rv3913	Rv3816c		Rv1187	

* predicted to be sncRNA-1 target by TargetRNA2

Table S3. Functions of DEG involved in fatty acid metabolism in *Mtb* 6230 overexpressing sncRNA-1 retrieved from Mycobrowser

up/down	gene name	function
up	desA2 (Rv1094)*	Thought to catalyze the principal **conversion of saturated fatty acids to unsaturated fatty acids**.
	accD6	Involved in **fatty acid biosynthesis** (mycolic acids synthesis)
	fabG4 (Rv0242c)*	Involved in the **fatty acid biosynthesis** pathway (first reduction step)
	fadD28	Involved in **phthiocerol dimycocerosate (dim) biosynthesis**. Thought to be involved in the release and transfer of mycoserosic acid from mas onto the DIOLS.
	fadA2	Function unknown, but involved in **lipid degradation**
	accA3	Involved in **long-chain fatty acid synthesis** (at the first step).
	fadE23	Function unknown, but involved in **lipid degradation**.
	rocA	Involved in the **production of acetyl-CoA** in the beta-ketoadipate pathway (at the third step)
down	fadE15	Function unknown, but involvement in **lipid degradation**.
	echA19	Enoyl-CoA hydratase (ECH) or crotonase is an enzyme that hydrates the double bond between the second and third carbons on 2-trans/cis-enoyl-CoA: ECH is essential to **metabolizing fatty acids in beta oxidation** to produce both acetyl CoA and energy in the form of ATP.
	hsaG	Supposed involvement in one, or several, **catabolic pathways** [catalytic activity: acetaldehyde + CoA + NAD(+) = acetyl-CoA + NADH]. Predicted to be involved in lipid catabolism.
	fadE29	Function unknown, but involved in **lipid degradation**.
	php	Probable phosphotriesterase Php, involved in **catabolic reactions**

* predicted to be sncRNA-1 target by TargetRNA2

Table S4. SncRNA-1 targets predicted using custom parameters in RNATarget2

Predicted target	Free energy	p value
RVBD_0196	-12.08	0.007
RVBD_0233	-10.36	0.019
RVBD_0242c*	-11.4	0.011
RVBD_0256c	-8.32	0.049
RVBD_0292	-16.43	0
RVBD_0364	-13.72	0.002
RVBD_0461	-14.86	0.001
RVBD_0499	-10.84	0.015
RVBD_0516c	-9.58	0.029
RVBD_0586	-9.14	0.035
RVBD_0807	-19.54	0
RVBD_0932c	-11.02	0.014
RVBD_1094*	-11.19	0.012
RVBD_1184c	-9.09	0.036
RVBD_1227c	-10.73	0.016
RVBD_1238	-8.71	0.042
RVBD_1264	-12.4	0.006
RVBD_1290c	-14.23	0.001
RVBD_1312	-8.52	0.046
RVBD_1420	-12.42	0.006
RVBD_1908c	-10.34	0.02
RVBD_1939	-8.81	0.04
RVBD_1953	-9.65	0.028
RVBD_2000	-9.72	0.027
RVBD_2090	-11.54	0.01
RVBD_2178c	-10.01	0.023
RVBD_2357c	-12.2	0.007
RVBD_2386A	-12.67	0.005
RVBD_2528c	-12.84	0.004
RVBD_2600	-8.31	0.05
RVBD_2737c	-11.42	0.011
RVBD_2883c	-13.76	0.002
RVBD_2937	-11.16	0.013
RVBD_3263	-9.84	0.025
RVBD_3444c	-9.87	0.025
RVBD_3511	-11.47	0.01
RVBD_3544c	-11.04	0.013
RVBD_3712	-9.27	0.033
RVBD_3742c	-11.11	0.013

* upregulated in sncRNA-1 vs ctl vector

Table S5. LNA-PIs, primers and plasmids used in this study

Primer	Description	Sequence
**target sequence for miRNA q-RT-PCR**	sncRNA-6	AAAGAACATGATGGGCGCACC
	sncRNA-4	GAAATGACGCAATGACCTCT
	sncRNA-8	ACTCAATAGTGTGTTTGGTGGTTT
	sncRNA-1	ACACGGGATCGGGCGAGTTCGACCT
	5S	GTCCCATTCCGAACCCGGAAGCTAAGCCTG
**target sequence for LNA-Pis**	sncRNA-6	GCCCATCATGTTCTTT
	sncRNA-8	ACCACCAAACACACTA
	sncRNA-1	TCGAACTCGCCCGATC
	negative control	TAACACGTCTATACGCCCA
**primers for q-RT-PCR**	5S qPCR FWD	ATT CCG AAC CCG GAA GCT AA
	5S qPCR REV	TTC GGC GGT GTC CTA CTT T
	16S qPCR FWD	AAG AAG CAC CGG CCA ACT AC
	16S qPCR REV	TCG CTC CTC AGC GTC AGT TA
	pre-sncRNA-1 qPCR FWD	TCG CGT AGA ATA GCG AAA CAC
	pre-sncRNA-1 qPCR REV	AGA GTG CGC TCA AAC GTA TAA A
	pre-sncRNA-6 qPCR FWD	AGC GCC CAT CAT GTT CTT T
	pre-sncRNA-6 qPCR REV	CGC ACA ACA GCA CCA ATA AC
	pre-sncRNA-8 qPCR FWD	TGA CTC CAT TGC CGG ATT T
	pre-sncRNA-8 qPCR REV	GTG AAA CCA CCA AAC ACA CTA TT
	rv1094 qPCR FWD	CGC GGT GTT CTG TCG TAA T
	rv1094 qPCR REV	GTA ACG AGG TTG GCG AAG AA
	rv0242c qPCR FWD	CTA CGA CCT GGT AGG AAA CAA C
	rv0242 qPCR REV	GCA GTA CCG GAG TAA AGA ACT C
**primers for cloning**	pka-303 FWD	gcttacataaacagtaatacTCAGGCGCCGGGGGCGGT
	pka-303 REV	aactcatcgagcatcaaatgGTGACACAAGAATCCCTGTTACTTCTCGACCGTA TTGATTCGGATGATTCCTACG
	hygroR FWD	CATTTGATGCTCGATGAGTTTTTCTAATCAGAATTGGTTAATTGGTT GTAACACTGG
	hygroR REV	GTATTACTGTTTATGTAAGCAGACAGTTTTATTGTTCATG
	pSUM-mcs1 FWD	GCA GGA CTG AAG CGG GAC TCT GGG GTT C
	pSUM-mcs1 REV	ACT TGG CCA TGC GAA ACG ATC CTC ATC CTG
	neoR FWD	ATC GTT TCG CAT GGC CAA GTT GAC CAG TG
	neoR FWD	GAG TCC CGC TTC AGT CCT GCT CCT CGG C
	Rv0242c_backbone_fwd	CAA CCC AGC GAA TTC GAG CTC GGT ACC C
	Rv0242c_backbone_rev	CCC CTG CTG CTA TGT TTT TCC TCC TTA TAA AGT TAA TCA G
	Rv0242c_fwd	GAA AAA CAT AGC AGC AGG GGC CAC TGT T
	Rv0242c_rev	AGC TCG AAT TCG CTG GGT TGA GTC ACT ACT G
	Rv1094_backbone_fwd	CGT CGG CGC GAA TTC GAG CTC GGT ACC C
	Rv1094_backbone_rev	CGT TGC GGT TTA TGT TTT TCC TCC TTA TAA AGT TAA TCA G
	Rv1094_fwd	GAA AAA CAT AAA CCG CAA CGC CGT CCC C
	Rv1094_rev	AGC TCG AAT TCG CGC CGA CGG GTG TCT AG
**Plasmid**	**Description**	**Selection**
**726**	pKa-303	kanamycin
**727**	pKa-kan-sncRNA-1	kanamycin
**739**	pKa-kan-sncRNA-6	kanamycin
**740**	pKa-kan-sncRNA-8	kanamycin
**880**	pKa-hyg-303	hygromycin
**881**	pKa-hyg-sncRNA-1	hygromycin
**882**	pKa-hyg-sncRNA-6	hygromycin
**883**	pKa-hyg-sncRNA-8	hygromycin
**671**	psum-kan-mcs1-gfp	kanamycin
**884**	psum-zeo-mcs1-gfp	zeocin
**885**	psum-zeo-mcs1-rv0242c	zeocin
**886**	psum-zeo-mcs1-rv1094	zeocin
**887**	pMyC	hygromycin
**888**	PMSG360zeo	zeocin
